# FLIM imaging revealed spontaneous osteogenic differentiation of stem cells on gradient pore size tissue-engineered constructs

**DOI:** 10.1186/s13287-023-03307-6

**Published:** 2023-04-12

**Authors:** Svetlana Rodimova, Artem Mozherov, Vadim Elagin, Maria Karabut, Ilya Shchechkin, Dmitry Kozlov, Dmitry Krylov, Alena Gavrina, Vladislav Kaplin, Evgenii Epifanov, Nikita Minaev, Ksenia Bardakova, Anna Solovieva, Peter Timashev, Elena Zagaynova, Daria Kuznetsova

**Affiliations:** 1 N. I. Lobachevsky Nizhny Novgorod National Research State University, 23 Gagarina Ave., Nizhny Novgorod, Russia 603022; 2 Institute of Experimental Oncology and Biomedical Technologies, Privolzhsky Research Medical University, 10/1 Minin and Pozharsky Sq., Nizhny Novgorod, Russia 603000; 3 Semenov Federal Research Center of Chemical Physics, Russian Academy of Sciences, 4 Kosygina St, Moscow, Russia 119991; 4 Research Center “Crystallography and Photonics”, Institute of Photonic Technologies, Russian Academy of Sciences, 2 Pionerskaya St, Troitsk, Moscow, Russia 108840; 5 Institute for Regenerative Medicine, Sechenov University, 8-2 Trubetskaya Str, Moscow, Russia 119991; 6 World-Class Research Center “Digital Biodesign and Personalized Healthcare”, Sechenov University, 8-2 Trubetskaya Str, Moscow, Russia 119991

**Keywords:** Heterogeneous scaffolds, MSC, Bone defects, Multiphoton microscopy, FLIM

## Abstract

**Background:**

There is an urgent clinical need for targeted strategies aimed at the treatment of bone defects resulting from fractures, infections or tumors. 3D scaffolds represent an alternative to allogeneic MSC transplantation, due to their mimicry of the cell niche and the preservation of tissue structure. The actual structure of the scaffold itself can affect both effective cell adhesion and its osteoinductive properties. Currently, the effects of the structural heterogeneity of scaffolds on the behavior of cells and tissues at the site of damage have not been extensively studied.

**Methods:**

Both homogeneous and heterogeneous scaffolds were generated from poly(L-lactic acid) methacrylated in supercritical carbon dioxide medium and were fabricated by two-photon polymerization. The homogeneous scaffolds consist of three layers of cylinders of the same diameter, whereas the heterogeneous (gradient pore sizes) scaffolds contain the middle layer of cylinders of increased diameter, imitating the native structure of spongy bone. To evaluate the osteoinductive properties of both types of scaffold, we performed in vitro and in vivo experiments. Multiphoton microscopy with fluorescence lifetime imaging microscopy was used for determining the metabolic states of MSCs, as a sensitive marker of cell differentiation. The results obtained from this approach were verified using standard markers of osteogenic differentiation and based on data from morphological analysis.

**Results:**

The heterogeneous scaffolds showed improved osteoinductive properties, accelerated the metabolic rearrangements associated with osteogenic differentiation, and enhanced the efficiency of bone tissue recovery, thereby providing for both the development of appropriate morphology and mineralization.

**Conclusions:**

The authors suggest that the heterogeneous tissue constructs are a promising tool for the restoration of bone defects. And, furthermore, that our results demonstrate that the use of label-free bioimaging methods can be considered as an effective approach for intravital assessment of the efficiency of differentiation of MSCs on scaffolds.

**Supplementary Information:**

The online version contains supplementary material available at 10.1186/s13287-023-03307-6.

## Background

Bone defects resulting from fractures, infections or tumors require targeted strategies for effective clinical treatment. Although bone tissue is capable of recovering small defects on its own, in the presence of extensive defects, as well as in cases of insufficient blood supply or the presence of systemic diseases, the regenerative potential of the bone tissue is violated [1]. Thus, the therapy of bone defects requires targeted strategies for effective clinical treatment.

Autologous bone, or autograft, is still considered the clinical “gold standard” and the most effective method for enabling bone regeneration, with its great osteoinductive and osteoconductive capabilities. Currently, this technique is still widely used; however, its disadvantages are also well known: the limitation on graft volume, donor site damage, differences in the structure and in the biomechanics of different skeletal parts, infection and immunological graft rejection [2–4]. Allogeneic bone grafts, also called allografts, are composed of materials taken from another individual. The osteoinductive capability of an allograft is dependent on the donor’s age, but is, in any case, minimal because of the low concentration of bone growth proteins as a result of the rigorous processes involved in the removal of potential antigenicity and pathogenicity. The fundamental problems of this grafting material are its antigenicity and the potential for the transmission of disease [5]. At the same time, the advantages of alloplastic grafts (scaffolds) include an absence of antigenicity, no potential for disease transmission, and their potentially unlimited supply. These constructs can be treated to be resorbable or non-resorbable and can be provided in various particle or pore sizes.

The main materials for bone-tissue engineering are bioceramics, metals, natural and synthetic polymers and various composites. Synthetic polymers offer possibilities for chemical modifications, molecular alterations and controlled biodegradation that facilitate tailoring of the system’s properties to the specific requirements of the application concerned. Poly(L-lactic acid) used in this current work undergoes hydrolytic degradation to form soluble lactic acid, which is present naturally in the body [3, 4]. Furthermore, cell-seeded scaffolds can provide improved osteoinductive properties and, in general, provide for better integration with the host tissues and remodeling than do acellular scaffolds [6].

The combination of a three-dimensional scaffold supplemented by growth factors and/or cells has great potential in bone tissue engineering as an ideal option for bone substitution.

Recently, the use of MSCs in regenerative medicine has been widely used because of their capacity to differentiate into multiple lineages, including osteogenic line. Early studies suggest that the micro-architecture of scaffolds might influence cell attachment and orientation as well as affecting cell fate [7, 8]. One of the main aspects of scaffold architecture is the pore structure, which is determined by the size, size distribution and geometry of the individual pores within the scaffold. Sufficient porosity of suitable size and interconnections between the pores, provide an optimal environment for promoting cell adhesion, migration, vascularization, proliferation and differentiation, as well as for maintaining effective nutrient and oxygen supply and the removal of wastes [9].

Previous studies have shown rapid bone formation is associated with pore sizes between 200 and 500 μm. In particular, smaller pore sizes enhance cell adhesion and the mechanical properties of the scaffold [10, 11]. Therefore, fabrication of a gradient porous scaffold, whereby the pore size changes from one layer to the next, may overcome some of the individual limitations of both small and large, homogeneous, pore-size scaffolds. Heterogeneous structures are suitable in providing for vascularization, the efficient diffusion of gases and nutrient supply. The crucial thing is that the heterogeneous scaffold architecture mimics the natural structure of native bone tissue that represents successively changing mineral density from cancellous bone to cortical bone [12, 13]. At the moment, there are very few studies showing the improved osteoinductive properties of heterogeneous scaffolds [14]. Furthermore, in these works only standard visualization methods were used, such as alkaline phosphatase (AP) activity evaluation and Alizarin Red S staining for the detection of mineralization. Unfortunately, these methods are invasive and do not allow long-term monitoring of the state of the cells seeded on the scaffolds.

Although the effect of scaffold structure on the metabolic state of cultured cells is still poorly understood, changes in this cell property could provide specific markers for the stage and efficiency of their differentiation.

More specifically, it is well known that many types of stem cells rely on glycolysis for energy, while in differentiated cells, oxidative phosphorylation (OXPHOS) becomes the main pathway for energy production [15, 16]. Therefore, a switch of energy source from glycolysis to OXPHOS can be considered as a hallmark of differentiated cells, including in the osteogenic direction [17–19]. In this connection, the approaches of metabolic imaging based on registration of the autofluorescence of NAD(P)H and FAD open up wide opportunities for studies of the cellular metabolic state. The ratio of the fluorescence intensities (FAD/NADH or FAD/(NADH + FAD), referred to as the optical redox ratio, is commonly used to estimate the metabolic state of a cell [20].

Although there are a significant number of studies of cellular energy metabolism based on optical redox ratio, this approach does not make it possible to separate the spectrally overlapping free and protein-bound forms of the cofactors, because the emission spectra are almost identical and differ only by 10–20 nm with a peak width of about 150 nm [21, 22].

Fluorescence lifetime imaging microscopy (FLIM) can overcome some of the challenges of intensity-based measurements as it provides the possibility to distinguish the free and protein-bound forms of NAD(P)H and FAD [23, 24]. By assessing the contributions of each form of NAD(P)H or FAD, it is possible to assess the prevalence of glycolysis or OXPHOS in cellular metabolism. Therefore, comprehensive information can be obtained by combining optical redox ratio measurements with FLIM data. Thus, the modern approach based on the registration of autofluorescence intensities and the fluorescence lifetimes of the NAD(P)H and FAD is a promising and effective tool for assessing the efficiency of osteogenic differentiation by examining the cellular metabolic state.

In our early studies, scaffolds made of methacrylated in supercritical carbon dioxide medium polylactide showed no cytotoxic effects [25].

The aim of our work has been to conduct a comparative analysis of the osteogenic differentiation and bone formation efficiency on both heterogeneous and homogeneous scaffolds using the new approach of metabolic imaging in combination with FLIM.

## Methods

### Scaffold fabrication

To obtain scaffolds, polylactide (Aldrich, Mw = 10–18 kDa) methacrylated by the urethane formation reaction in a supercritical carbon dioxide medium was used. Ethylene glycol methacrylic ether acted as a carrier of unsaturated groups, and isophorone diisocyanate was used as a linker in the urethane formation reaction. The photoactive composition was a 15% polylactide methacrylate solution in methylene chloride containing a cross-linking agent—20% wt of polylactide oligourethanedimethacrylate (to provide linking groups to form a three-dimensional cross-linked mesh)—and a photoinitiator (Michler’s ketone, 0.5% wt of polylactide). The composition (in the form of a viscous liquid) was placed using layer-by-layer deposition on a coverslip. The second harmonic of a femtosecond laser with a wavelength of 525 nm was used for the formation of the three-dimensional structures by two-photon polymerization. The photosensitive composition is transparent to laser radiation; however, a nonlinear process that consists of the simultaneous absorption of two photons by the photoinitiator molecule may occur in the focusing area in the case of the high power density required by the ultralow duration of the impulse. This corresponds to the absorption of a single photon at a wavelength of 263 nm in the focus area. The layers were formed with the aid of a galvo scanner. The travel between the layers was accomplished with the use of the Z-stage, which moved the galvo scanner with the objective along the vertical axis. Thus, the entire volume of the three-dimensional model was filled with appropriately assigned layers to produce a bulk cylinder from the polymerized composition within the source material. The cross-linked scaffolds were kept in tetrahydrofuran to remove any unreacted polymer and dopants and then dried in air [25–27].

### Scaffold characteristics

The scaffold structures represent a 3-layer array consisting of hexagonally stacked cylinders, fabricated using two-photon polymerization [25]. The homogeneous structures were produced of cylinders with an inner diameter of 180 microns, an outer of 250 microns and a height of 80 microns [25]. The heterogeneous structures contained an containing enlarged cylinders central pore-layer of cylinders with an inner diameter of 300 microns, an outer of 400 microns and a height of 100 microns (see Additional file 1: Fig. S2).

A 3D model of a scaffold with a heterogeneous structure is shown in Additional file 1: Fig. S1. The mechanical properties of these scaffolds were determined by an indentation technique using the Mach-1 v500csst Micromechanical Testing System (Biomomentum Inc., Laval, QC, Canada). The indentation was performed with a spherical indenter (1 mm diameter) at 0.05 mm/s speed and a maximum load force of 1 N. At least 5 measurements were collected per sample at different points. The Hertz’s contact model, corrected for the sample thickness, was applied:

$$F=f(\delta )\frac{4}{3}\sqrt{R}\frac{E}{(1-{\upsilon }^{2})}{\delta }^\frac{3}{2}$$

where *F* is the force acting on the indenter; *δ* is the indentation depth; *υ* is the Poisson’s ratio of the sample (assumed to be 0.33 for the studied material); *R* is the radius of the indenter; $f(\delta )$ is the thickness correction factor [28]; E is the effective sample Young’s modulus.

### In vitro model

#### Cell culture

Human bone marrow-derived MSCs were isolated from the bone marrow of healthy donors, with their informed consent according to the institutional guidelines under the approved protocol. Mononucleate cells were separated by centrifugation over a Histopaque gradient (#10771, Sigma-Aldrich, St. Louis, MO, USA), suspended in regular growth medium -Dulbecco’s modified Eagle’s medium, or DMEM (#11966025, Gibco, USA) supplemented with 10% fetal bovine serum (FBS) (#A3160501, Gibco, USA), 0.58 mg/mL L-glutamine (#F032 PanEco, Russia), and 40 U/mL gentamicin (#A011p, PanEco, Russia), and plated on culture flasks. After 3 days, non-adherent cells were removed by washing with phosphate-buffered saline (PBS), and monolayers of adherent cells were cultured until they reached confluence. The cells were then trypsinized (0.25% trypsin with 0.1% EDTA) (# P034, PanEco, Russia) and subcultured.

#### Experimental groups

The experimental stage included four groups of samples: homogeneous/ heterogeneous scaffolds, cultured in DMEM medium; homogeneous/ heterogeneous scaffolds cultured in a medium for the induction of osteogenic differentiation.

**Fig. 1:**
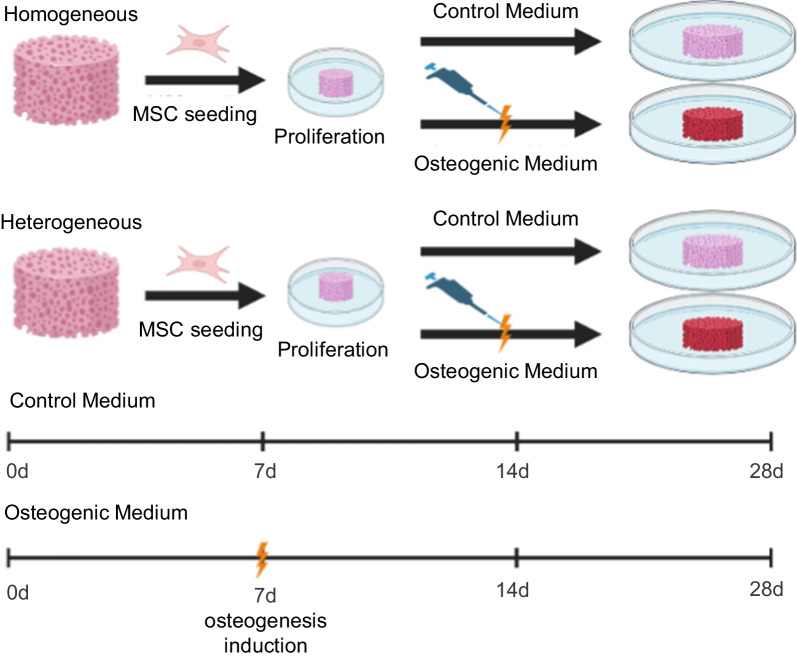
Road map of the steps of the osteogenic differentiation protocol for the in vitro model

The MSCs were cultured in MesenCult™ MSC Basal Medium (Human) (#05401, Stemcell Technologies, Canada) supplemented with 10% FBS, 0.58 mg/ml L-glutamine (#A011p, PanEco, Russia) and the antibiotic–antimycotic—100 unit/mL of penicillin, 100 μg/mL of streptomycin, 25 μg/mL of Fungizone™ (#15240062, Gibco, USA). The cell culture was maintained at 37 °C in a 5% CO^2^, humidified atmosphere. On the 3rd day, the MSCs were seeded onto the sterilized scaffolds. Each scaffold was inoculated with 20 μL of cell suspension (3 × 10^5 cells) on the surface of the top layer of the scaffold. Next, the scaffolds were incubated under the standard conditions (37 °C, 5% CO2, saturation humidity) for 90 min before 1 mL of DMEM medium was carefully added to each well containing the seeded scaffolds. Differentiation was induced by incubating the MSCs in MesenCult™ Osteogenic Stimulatory Kit (Human) (# 05465, Stemcell Technologies, Canada) according to the manufacturer's protocol. The medium was replaced every 3–4 days over the experimental period of up to 28 days. The scheme of the experiment is shown in Fig. 1.

#### Cell viability

For the analysis of cell viability, a mixture of calcein and propidium iodide was prepared according to the manufacturer's protocol (#04511-1KT-F, Live/Dead Cell Double Staining Kit, Thermo Fisher Scientific, USA). The samples were washed with PBS and stained with the prepared mixture. Immediately before the study, the dye was replaced by FluoroBrite DMEM Medium (#A1896701, Gibco, USA). Additionally, the cell nuclei were stained with Hoechst fluorescent dye (#O160, PanEco, Russia). Microscopic examination was carried out on an LSM 880 (Zeiss) using a C Plan-Apochromat 40x/1.3 oil-immersion objective. Images were acquired with a resolution of 1024 × 1024 pixels and averaged over two scans. Visualization was performed with the following parameters of excitation and registration: calcein—488 nm and 500–570 nm, propidium iodide—543 nm and 600–700 nm and Hoechst—405 nm and 470–510 nm, respectively. For each image, we obtained 10 fields of view. For each field of view, we counted the number of living and dead cells.

#### Effectiveness of osteogenic differentiation

Osteogenic differentiation was verified by staining calcifications of the extracellular matrix with Alizarin Red S according to the manufacturer's protocol (#130-22-3, Sigma-Aldrich, USA). The analysis was carried out visually over the entire surface of the scaffolds using a Leica DM 2500 microscope (Leica, Germany). Quantification of the mineralization of scaffolds stained with Alizarin Red S was performed using ImageJ software. For each scaffold, we calculated the percentage of the stained area to the total area in each of 10, fields of view × 100.

We used the Alkaline Phosphatase (AP) commercial Kit for fluorometric AP activity assay, according to the manufacturer's protocol (#APF-1KT, Sigma-Aldrich, USA). Analysis of the AP content was performed using a plate reader (Synergy MX, BioTek) at 360 nm excitation and 440 nm emission. As the enzyme activity increases with time, the plate was read three times with an interval of 5 min. Three wells of each sample (medium from homogeneous scaffolds, medium from heterogeneous scaffolds, and medium without scaffolds) were used as an internal control without the addition of substrate.

#### Real-time PCR

Isolation of total RNA from the samples was performed according to the protocol for the Direct-zol DNA/RNA Miniprep kit. Before the reverse transcription reaction, the samples were treated using a TURBO DNA-free™ Kit (#AM1907, Invitrogen, Carlsbad, California, USA). Real-time PCR was performed using a CFX96 Real Time PCR (Bio-Rad, Hercules, CA, USA) system involving a SYBR Green dye-based PCR amplification assay (#7567, Molecular Probe, USA). The PCR contained 1-x GeneAmp PCR Buffer I (#8,080,129, Applied Biosystems, Waltham, Massachusetts, USA), 250 µM of each dNTP, 0.5 nM of each primer (primer sequences are shown in Additional file 1: Table S1), and 1 U of Taq M polymerase (#751–100, Intifica, Saint Petersburg, Russia); the total concentration of Mg2 + in the reaction was 3 mM and the reaction volume was 20 µL. Temperature profile of cycles: 1) 95 °C for 10 min (enzyme activation step); 2) 35 cycles of 95 °C for 15 s, 60 °C for 30 s, and 72 °C for 30 s; 3) hybridization—95 °C for 1 min and 40 °C for 1 min; 4) melt curve analysis with measurements between 60 and 95 °C. The reaction efficiency was determined using a calibration curve method. Quantitative RT-PCR analysis was performed using CFX Maestro 2.3 software. HPRT1 was used as a reference gene.

#### Multiphoton fluorescence microscopy and FLIM

Using multiphoton microscopy, we analyzed the metabolic state of the MSCs on scaffolds with heterogeneous and homogeneous architectures during long-term cultivation in both DMEM (control medium) and osteogenic medium. Investigation of all samples was performed using a LSM 880 (Carl Zeiss, Germany), equipped with a Ti:Sapphire femtosecond laser (repetition rate: 80 MHz, a pulse duration less than 100 fs) and a time-correlated single-photon counting (TCSPC) system, Simple-Tau 152 (Becker & Hickl GmbH, Germany). The average power at the samples was about 10 mW. An oil immersion objective C Plan-Apochromat 40x/1.3 was used to collect the fluorescence signal. For each sample, 10 autofluorescence images of NAD(P)H and FAD were obtained. Fluorescence intensity images of 1024 × 1024 pixels and 212 × 212 µm, plus FLIM images of 512 × 512 pixels were acquired from the same fields of view. Using the ImageJ (integrated density parameter), we obtained the values of the autofluorescence intensity of the NAD(P)H and FAD, as well as values of the optical redox ratio FAD/NAD(P)H. NAD(P)H: excitation—750 nm, emission—455–500 nm; FAD: excitation—900 nm, emission—500–550 nm. FLIM microscopy was used to obtain data on the fluorescence lifetimes and their contributions of the various forms of NAD(P)H and FAD. The optical redox ratio was defined as the fluorescence intensity of FAD divided by the fluorescence intensity of NAD(P)H. The intensity of NAD(P)H and FAD autofluorescence, as well as the redox ratio, was calculated from corresponding two-photon fluorescence images of FAD and NAD(P)H after subtracting the background on a pixel-by-pixel basis using ImageJ 1.39p software (NIH, Bethesda, MD, USA). The fluorescence lifetime data were analyzed using the SPCImage program (Becker & Hickle GmbH, Germany). To maintain a minimum of 5000 counts per pixel, the binning parameter was set at 3. The goodness-of-fit model was assessed by *χ*2 value. (It should be at 1.) The FLIM analysis was performed using SPCImage software with 20 ROIs being analyzed in each image to evaluate the following parameters: t1 (ps) is the fluorescence lifetime of the short component (free form NAD(P)H and the closed form FAD); t2 (ps) is the fluorescence lifetime of the long component (the bound form of NAD(P)H and the open form of FAD); a1 (%) is the contribution of the short component; a2 (%)—contribution of the long component. To reduce the contribution of the background fluorescence from the scaffold, cells were manually selected from the centers of the pores of the scaffolds. For each image, we selected the optimal threshold parameter using the SPCImage program.

### In vivo model

#### Animal model

For in vivo analysis, seeded scaffolds planted with cells according to the method described above were implanted into mice. Mouse bone-marrow-derived MSCs were isolated from the marrow of the diaphysis of mouse femur and tibia under the approved protocol [29]. Each scaffold was inoculated with 20 μL of cell suspension (3 × 10^5 cells) on the surface of the top layer of the scaffold. Next, the scaffolds were incubated under the standard conditions (370C, 5% CO2, saturation humidity) for 90 min, before 1 mL of DMEM medium was carefully added to each well with scaffolds and cultured for 3 days. Before implantation, we assessed the metabolic state of the MSCs using the FLIM method, based on data on the lifetimes of NAD(P)H and FAD and their relative contributions to the MSCs’ autofluorescence. To demonstrate the cells' viability before implantation into the mice, several scaffolds with MSCs were stained with the specific fluorescent dyes: Calcein/Propidium Iodide (#04511-1KT-F, Live/Dead Cell Double Staining Kit, Sigma-Aldrich, USA). First, an assay solution was prepared according to the Sigma-Aldrich protocol. Then, the scaffolds carrying MSCs were washed with PBS, placed into the assay solution and incubated at 37 °C for 20 min. After the staining, the fluorescence of the cells was detected using a fluorescence microscope.

The experiments were performed on wild-type male C57/Bl6 mice, at the age of two months, weighing 20 g. The animals were divided into two groups—with implanted homogeneous and heterogeneous scaffolds (n = 15, for each group). The mice were anesthetized with Zoletil at a concentration of 80 mg/kg. Non-healing, full-thickness defects 4 mm in the diameter were made in the cranial bone, while critical-size defects were created on the calvarial bone of each animal using a dental bur. Immediately after the generation of the cranial bone defects, the scaffolds with seeded cells were implanted into the injury sites. After that, the skin patch was closed, and sutures inserted. The cranial bones with implanted scaffolds of both types were harvested and assayed at the 4th, 8th and 12th weeks after scaffold implantation. Animals were removed from the experiment by dislocation of the cervical vertebrae. All experiments were carried out according to the ARRIVE guidelines for of animal experiments.

**Fig. 2:**
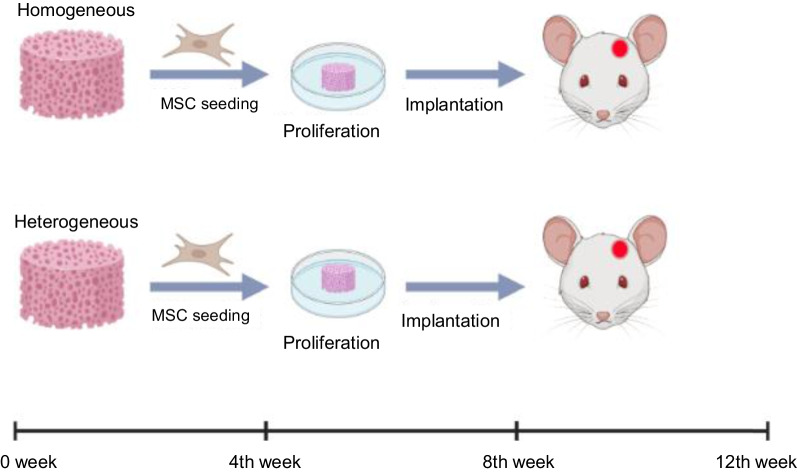
Road map of the steps of the scaffold implantation protocol for the in vivo model

The scheme of the experiment is shown in Fig. 2.

#### Bone formation analysis after implantation of scaffolds in mice

Scaffold biodegradation was analyzed in vivo using light microscopy on a stereo zoom microscope (Axio Zoom V16, Carl Zeiss, Germany), field of view 9 × 12 mm. Then, to analyze the state of the cells seeded on the scaffolds using multiphoton microscopy and FLIM, the cranial fragments were harvested.

#### Histology

The cranial bones were fixed in 10% formalin solution and decalcified with nitric acid for 2 weeks. Then, the samples were dehydrated in an ascending ethanol series before being embedded in paraffin wax. 5 µm sections were cut using a microtome (Leica SM 2000; Leica, Germany) and mounted on glass slides. Ten cross-sections from the middle of each implant were stained with hematoxylin and eosin and Van Gieson.

### Statistical analysis

Statistical analysis of the expression genes related to osteogenesis were calculated using the Mann-Whitney test. To analyze the metabolic state of the MSCs using FLIM, 20 regions of interest (ROIs) in the cell cytoplasm were studied for each image. The mean values and the standard deviation (SD) were calculated for each of the investigated parameters. Scatter plots were constructed to analyze the distribution of the FLIM parameters for all groups in the study. The middle line of the boxplot is the median, the poiborders represent the interquartile range, each point corresponding to the value of the presented FLIM parameter for an individual cell. Statistical analyses were conducted by STATISTICA 10 (StatSoft Inc., Tulsa, OK, USA) using the ANOVA Bonferroni test.

## Results

### Scaffold mechanical properties

The scaffolds had an effective Young’s modulus of 140 ± 40 MPa as measured with the indentation technique. An example of the load-indentation curve is presented in Additional file 1: Fig. S2. The indentation with a 1-mm-diameter probe provides the effective modulus of the scaffold itself rather than of the material from which it is made (typical modulus is in the order of 1 GPa) [5]. Thus, considering the porous structure of the scaffold, the lower, effective Young’s modulus is expected. Yet, the modulus is high enough to preserve the structural integrity of the scaffold.

### In vitro model

#### Cellular morphology and proliferation

**Fig. 3:**
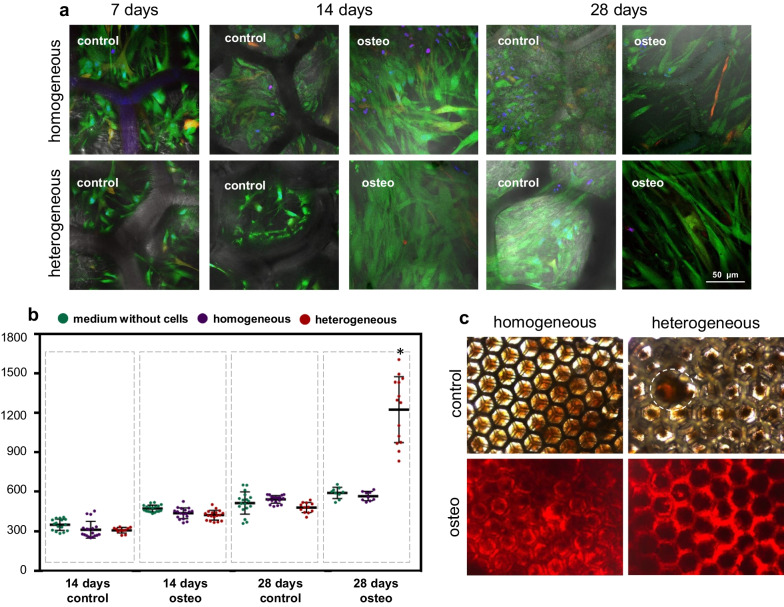
**a** Fluorescent images of MSCs on scaffolds stained with calcein and propidium iodide, cell nuclei stained with Hoechst; × 400; **b** analysis of alkaline phosphatase activity in culture media obtained during cultivation in DMEM and in a medium for the induction of osteogenic differentiation; **c** staining with Alizarin Red S for the detection of calcifications in MSCs on homogeneous and heterogeneous scaffolds by the 28th day of cultivation in DMEM and in osteogenic medium; × 100. *statistically significant differences compared to the corresponding time point for MSCs on homogeneous scaffolds; Mean ± SD, *p*-value ≤ 0.05

MSCs cultured on scaffolds in DMEM had a polygonal shape, while MSCs on scaffolds cultured in the osteogenic medium were spindle-shaped. It should be noted that on homogeneous scaffolds, both in DMEM and in the osteogenic medium, the MSCs had a smaller size, than on heterogeneous scaffolds, which may be due to the higher cell density (Fig. 3a).

Analysis of the cell viability showed that on the 7th day the percentage of dead cells on the heterogeneous scaffolds was higher (3.7%) than on the homogeneous scaffolds (0.18%). By contrast, on the 14th day of cultivation in DMEM, the percentage of dead cells on the heterogeneous scaffolds was 0.29%, compared with the homogeneous scaffolds at 0.45%. In the case of cultivation in osteogenic medium, the proportions of dead cells on the heterogeneous scaffolds on 14th and 28th days were also significantly lower, than on the homogeneous scaffolds on the corresponding days of monitoring (5.84% and 1.07% for homogeneous, and 0.97% and 0.2% for heterogeneous) (Fig. 3a, Additional file 1: Table S2).

#### Evaluation of the effectiveness of osteogenic differentiation by standard methods

The analysis of the AP activity showed that the fluorescence signal from the DMEM after the MSCs had been cultivated on the scaffolds, on both the 14th and the 28th days, did not differ significantly from the signal in the DMEM without scaffolds (Fig. 3b).

These results indicate the absence of AP secretion. In the case of the osteogenic medium, there were no differences between the medium with and without scaffolds on the 14th day. However, by the 28th day, there was a significant increase in the value of the fluorescence signal in the osteogenic medium obtained after heterogeneous scaffold cultivation. At the same time, for the medium from the homogeneous scaffolds the values of the fluorescence signal remained similar to the background fluorescence in the DMEM without scaffolds (Fig. 3b).

Analysis of mineralization showed no spontaneous differentiation on the homogeneous scaffolds in DMEM by day 28. At the same time, for heterogeneous scaffolds cultured in DMEM we revealed diffusely located areas of mineralization, which confirmed the better osteoinductive properties of the heterogeneous scaffolds (Fig. 3c). In the case of the osteogenic medium, both types of scaffolds had extensive mineralization zones by the 28th day.

A quantitative assessment of the mineralization of scaffolds stained with Alizarin Red S showed that the level of mineralization for heterogeneous scaffolds cultured in DMEM was statistically significantly higher in comparison with the homogeneous scaffolds (*p*-value ≤ 0.05). Scaffolds cultured in DMEM: homogeneous 2.5 ± 0.89 and heterogeneous 18.26 ± 4.74. For scaffolds cultured in an osteogenic medium, there were also differences: homogeneous 89.44 ± 10.89 and heterogeneous 97.83 ± 3.49. However, although it was shown that the level of mineralization for heterogeneous scaffolds was higher, this was not statistically significant different from the homogeneous scaffolds.

Results obtained by standard methods confirmed the better osteoinductive properties of the heterogeneous scaffolds. Thus, heterogeneous scaffolds demonstrated both an increased AP activity in the osteogenic medium, and the presence of spontaneous differentiation even in the DMEM (control medium).

#### Multiphoton microscopy and FLIM

**Fig. 4:**
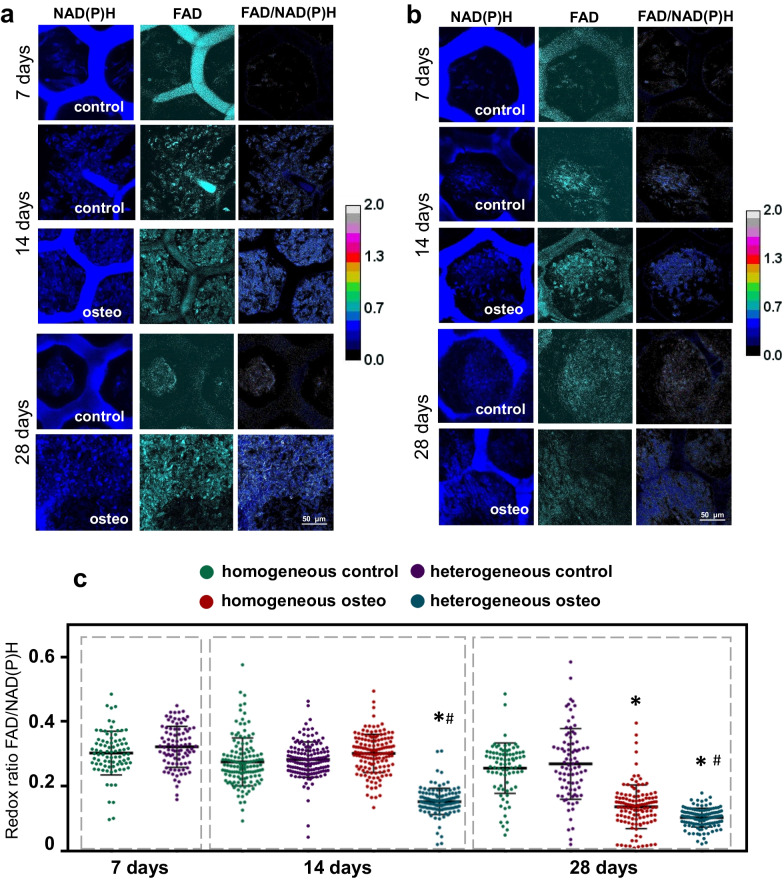
Autofluorescence images and images of the optical redox ratio of NAD(P)H and FAD in MSCs, during their cultivation on **a** homogeneous and **b** heterogeneous scaffolds; **c** scatter plots reflecting the distribution of the values of the optical redox ratio FAD/NAD(P)H; × 400. *statistically significant differences compared to the corresponding time point for the MSCs on scaffolds cultivated in DMEM; #statistically significant difference in MSCs on heterogeneous scaffolds cultivated in osteogenic medium compared to the corresponding time point for MSCs on homogeneous scaffolds cultivated in osteogenic medium; Mean ± SD, *p*-value ≤ 0.05

For all experimental groups, we revealed an increase in the intensity of NAD(P)H autofluorescence, which was most pronounced in the case of the osteogenic medium (Fig. 4a, b, Additional file 1: Table S3). The intensities of FAD autofluorescence in cells on scaffolds cultured in the osteogenic medium were higher compared to cells cultured in DMEM (Fig. 4a, b, Additional file 1: Table S3). The optical redox ratio FAD/NAD(P)H dropped significantly during the osteogenic differentiation for both types of scaffolds. The most pronounced decrease was in the MSCs on heterogeneous scaffolds. However, in cells on scaffolds cultured in DMEM, the optical redox ratio values remained stable throughout the entire cultivation period (Fig. 4a–c, Additional file 1: Table S3).

Thus, we can conclude that MSCs on scaffolds cultured in an osteogenic medium had a higher metabolic activity in comparison with cells on scaffolds cultured in DMEM. Comparison of the two types of scaffold structures revealed that during osteogenic differentiation the redox ratio FAD/NAD(P)H for cells on the homogeneous scaffolds on the 14th day was similar to the cells on scaffolds cultured in DMEM, while in MSCs on heterogeneous scaffolds, this parameter was significantly lower by the 14th day in comparison with cells cultured in DMEM. These results indicate a faster rate of metabolic rearrangements associated with osteogenic differentiation in the cells on heterogeneous scaffolds.

**Fig. 5:**
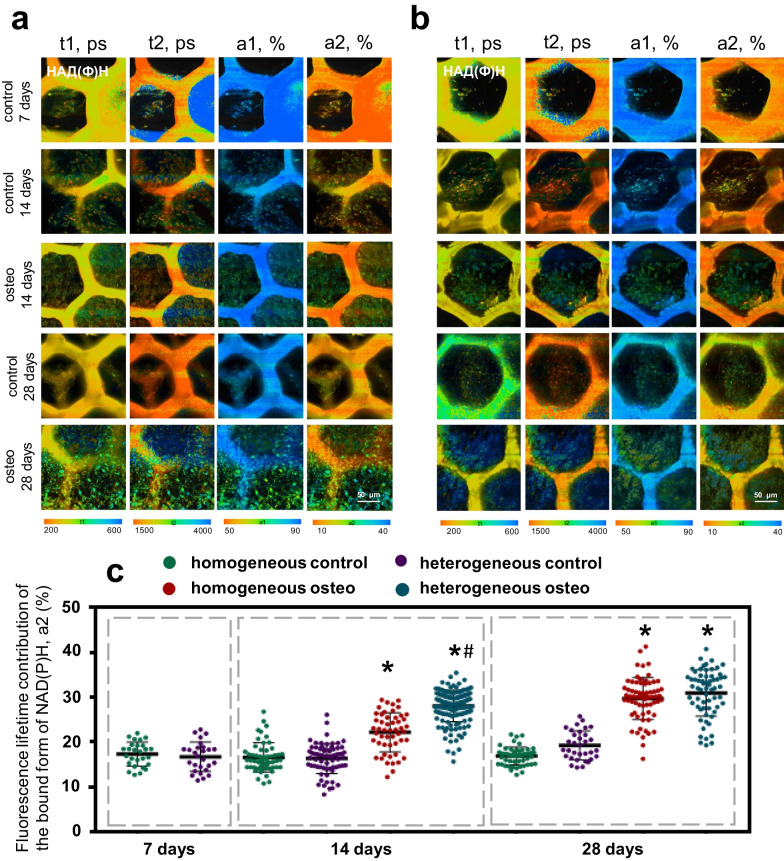
FLIM images of NAD(P)H for MSCs on **a** homogeneous and **b** heterogeneous scaffolds; **c** The values of the fluorescence lifetime contribution of the bound form of NAD(P)H during their cultivation in DMEM and in the medium for the induction of osteogenic differentiation; × 400. *statistically significant differences compared to the corresponding time point for the MSCs on scaffolds cultivated in DMEM; #statistically significant difference in MSCs on heterogeneous scaffolds cultivated in osteogenic medium compared to the corresponding time point for MSCs on homogeneous scaffolds cultivated in osteogenic medium; Mean ± SD, *p*-value ≤ 0.05

*NAD(P)H:* Analysis of FLIM data showed that in cells on scaffolds cultured in an osteogenic medium, there was a gradual increase in the fluorescence lifetimes of the free and bound forms of NAD(P)H (t1, t2) (Additional file 1: Table S4). In addition, in cells on scaffolds cultured in an osteogenic medium, we observed a gradual growth in the contribution of the bound form of NAD(P)H (a2, %), which indicated a shift in the metabolic state of the MSCs toward the prevalence of oxidative phosphorylation (OXPHOS) (Fig. 5a–c, Additional file 1: Table S4). Active OXPHOS is likely required to meet the high ATP demands of the extensive biosynthesis of extracellular matrix protein during osteogenesis.

The crucial point is that MSCs on homogeneous scaffolds at the early stages of osteogenic differentiation showed a smaller contribution of the bound form of NADH (a2) compared to the MSCs on heterogeneous scaffolds.

In the case of cultivation in DMEM, there were no changes in the fluorescence lifetimes of the free and bound form of NADH (t1, t2) during the entire cultivation period. The values of the contributions a1 and a2 also did not differ at the early stages of cultivation. However, on the 28th day, we observed a tendency towards an increase in the a2 values for MSCs on the heterogeneous scaffolds.

Such results confirm our findings based on the autofluorescence intensity of the cofactors and indicate an accelerated process of MSC differentiation and the greater osteoinductive properties of the heterogeneous scaffolds.

*FAD* The fluorescence lifetimes of the open and closed forms of FAD (t1, t2) in general remained stable throughout the entire cultivation period for all the studied groups. An exception was a slight decrease of t2 in the MSCs on homogeneous scaffolds on the 28th day of cultivation, probably associated with a decrease in the metabolic activity of the cells due to their high density (Additional file 1: Table S5).

**Fig. 6:**
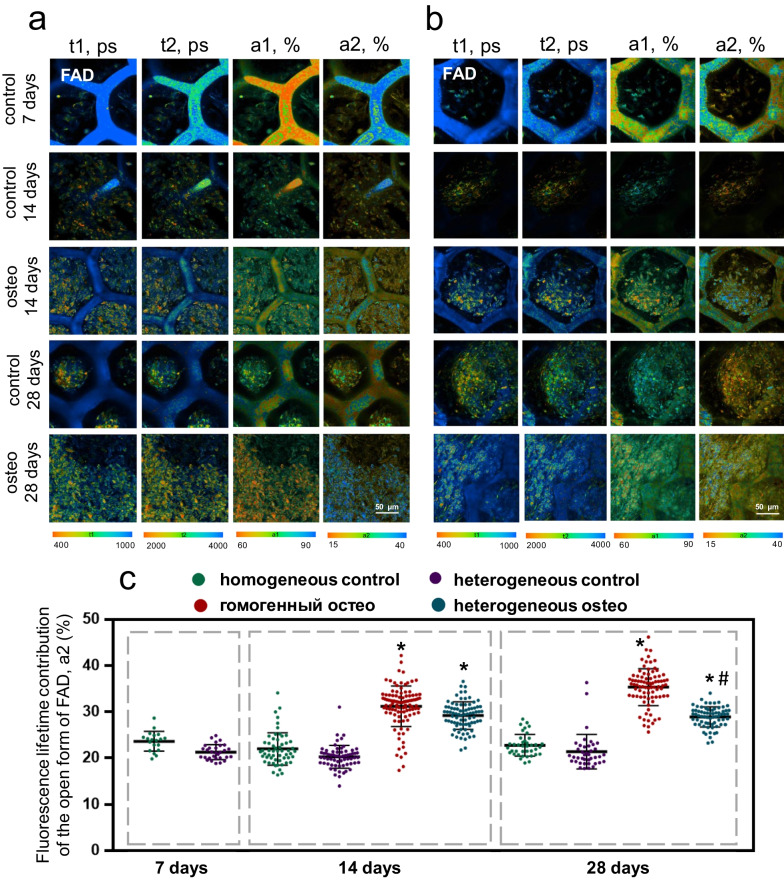
FLIM images of FAD for MSCs on **a** homogeneous and **b** heterogeneous scaffolds; **c** The values of fluorescence lifetime contribution of the open form of FAD during cultivation in DMEM and in osteogenic medium; × 400. *statistically significant differences compared to the corresponding time point for the MSCs on scaffolds cultivated in DMEM; #statistically significant difference in MSCs on heterogeneous scaffolds cultivated in osteogenic medium compared to the corresponding time point for MSCs on homogeneous scaffolds cultivated in osteogenic medium; Mean ± SD, *p*-value ≤ 0.05

We observed a growth in the contribution of the open form of FAD in cells on both types of scaffolds cultured in osteogenic medium (Fig. 6a–c, Additional file 1: Table S5), which confirmed the previously shown increase in intensity of OXPHOS during osteogenic differentiation, since FAD takes part in complex II of the mitochondrial electron transport chain.

In the case of MSCs on scaffolds cultivated in DMEM, the fluorescence lifetimes of the open and closed forms of FAD (t1, t2) and their contributions (a1, a2) remained stable throughout the entire cultivation period (Fig. 6a–c, Additional file 1: Table S5).

The FLIM data (for both NAD(P)H and FAD) confirmed that the greater osteoinductive properties of heterogeneous scaffolds are consistent with the results of the analysis of AP activity and the assessment of mineralization by Alizarin Red S.

#### Real-time PCR

In cells on heterogeneous scaffolds, during the later stages of cultivation in the control medium, higher levels of expression of the osteogenesis markers RUNX2 and SPARC were shown, in comparison with those in the cells on homogeneous scaffolds; levels of SDF-1 (a marker of undifferentiated MSCs) for both types of scaffolds gradually increased during the entire cultivation period, indicating the presence of actively proliferating undifferentiated MSCs (Fig. 5a). At the late stages of osteogenic differentiation, SDF-1 expression was low, and no significant differences in its expression level between the MSCs on homogeneous and heterogeneous scaffolds were found; the level of the osteogenesis marker COL1A1 gradually increased in cells on both types of scaffold, while no changes in RUNX2 expression were detected (Fig. 5b). More detailed information on the results of the molecular analyses is presented in Supplementary Materials (Additional file 1: Fig. S5).

### In vivo* model*

#### Biodegradation of scaffolds after implantation

**Fig. 7:**
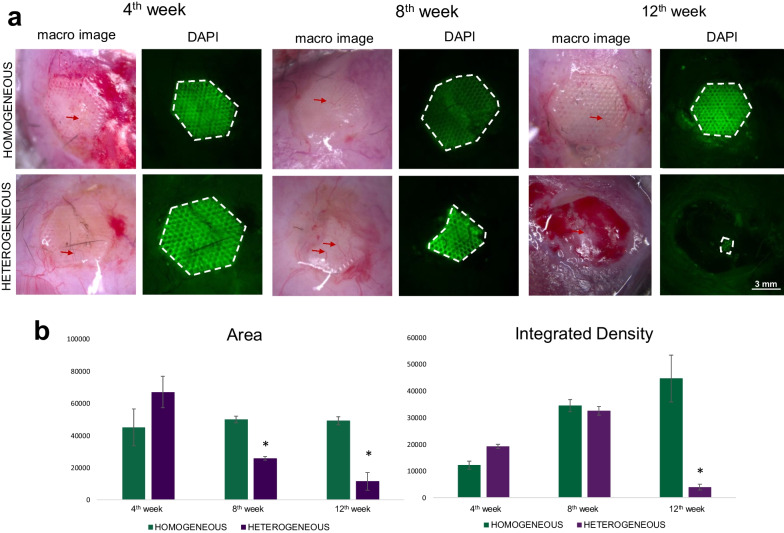
Scaffold biodegradation analysis using fluorescence stereo ZOOM microscopy. **a** Macro-images of implanted scaffolds with either a homogeneous or heterogeneous structure, obtained from the surface of the cranial bone of mice in vivo; the dotted lines mark the boundaries of the scaffolds; the red arrow indicate the formation of new blood vessels; **b** analysis of scaffold biodegradation based on the scaffold autofluorescence signal in the DAPI channel. The values of the area parameter and the integrated density parameters are presented in arbitrary units. *statistically significant differences in MSCs on heterogeneous scaffolds in comparison with MSCs on homogeneous scaffolds at corresponding time points; Mean ± SD, *p* < 0.05

Using a fluorescence stereomicroscope, we determined structural changes, as well as evaluating the rate of scaffold biodegradation after implantation into a cranial bone defect. Visual assessment showed that the rate of biodegradation for heterogeneous scaffolds was significantly higher compared to the homogeneous scaffolds (Fig. 7a).

Figure 7a shows that the area of homogeneous scaffolds did not change significantly at any of the chosen time points after implantation. Meanwhile, for the heterogeneous scaffolds, we revealed a significant decrease in their area by the 12th week, which indicated a high rate of biodegradation of these heterogeneous scaffolds (Fig. 7a). Also, using stereomicroscopy, we visualized the vessels in the defect zone with implanted scaffolds. For both types of scaffolds, vascular invasion into the zone of the bone defect could already be detected by the 4th week after scaffold implantation, indicating a high level of vascularization of both types of scaffolds (Fig. 7a). However, it should be noted that the level of vascularization for the heterogeneous scaffolds was higher.

Such results are also confirmed by analysis of the “Area” and “Integrated Density” parameters, where we observed a decrease in the Area parameter in the case of heterogeneous scaffolds (Fig. 7b). By contrast, the values of the “Area” were stable for homogeneous scaffolds throughout the experiment, indicating a low rate of biodegradation of the homogeneous scaffolds (Fig. 7b). The “Integrated Density” gradually increased for the homogeneous scaffolds at all studied time points, which may be due to a growth in the intensity of cellular autofluorescence during bone tissue formation. We revealed that the “Integrated Density” of the scaffolds increased by the 8th week for homogeneous scaffolds, possibly resulting from an increase in the autofluorescence intensity of the cells on the scaffolds. The same trend was observed for the homogeneous scaffolds up to the 8th week, however, at the 12th week there was a sharp decrease in the values of “Integrated Density”, which we associated with the decrease in the total area of the scaffold.

#### Metabolic imaging of scaffolds after implantation

Analysis of the metabolic state of MSCs on scaffolds of the two types was carried out specifically in the areas of the remaining undegraded scaffold, since it was rather difficult to carry out analysis using bioimaging methods in the formed bone tissue due to its high degree of mineralization.

We analyzed the metabolic state of the MSCs on the scaffolds before implantation (after 2 days of cultivation) and at different time points after implantation. Analysis of the FAD/NAD(P)H redox ratio in the MSCs on scaffolds before implantation showed a stable metabolic state of the cells on both the homogeneous and heterogeneous scaffolds (Additional file 1: Fig. S3). The average values of the redox ratio of FAD/NAD(P)H were 0.25 ± 0.02 for the homogeneous and 0.3 ± 0.01 for the heterogeneous scaffolds, reflecting the initially high level of cellular metabolic activity. In addition, we analyzed the viability of the MSCs before implantation (Additional file 1: Fig. S3). The number of dead cells on both types of scaffolds was insignificant (less than 2%). Using FLIM, it was shown that the fluorescence lifetimes of the free and bound forms of NAD(P)H in the MSCs for both types of scaffold had typical values (Additional file 1: Fig. S4) [30]. The fluorescence lifetime contributions of the free and bound forms of NAD(P)H and FAD in MSCs for both types of scaffolds also had typical values (Additional file 1: Fig. S4) [31]. In general, it can be concluded that the MSCs on the scaffolds before implantation were viable and metabolically active.

**Fig. 8:**
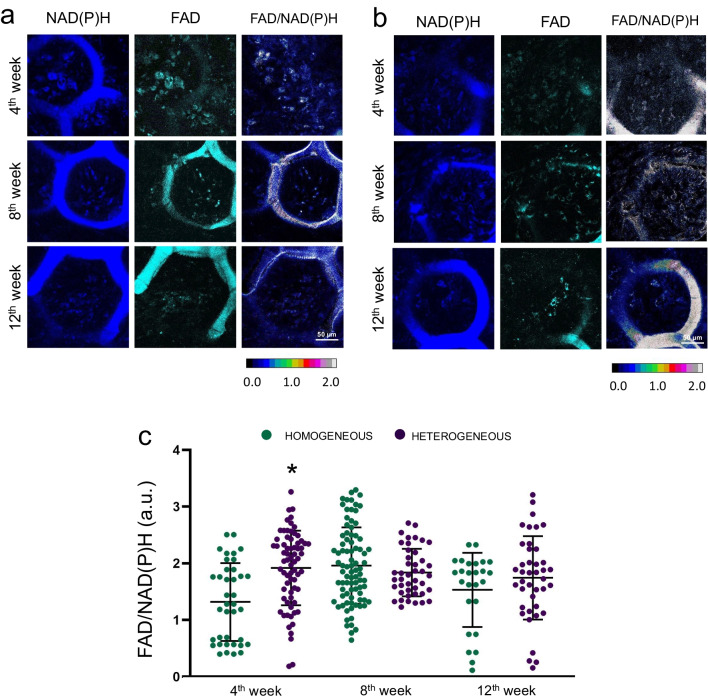
Autofluorescence and optical redox ratio images of NAD(P)H and FAD in MSCs on **a** homogeneous and **b** heterogeneous scaffolds after implantation. Field of view 213 × 213 µm (1024 × 1024 responses); × 400; **c** FAD/NAD(P)H redox ratio values in MSCs on homogeneous and heterogeneous scaffolds after implantation into the cranial bone. *statistically significant differences in MSCs on heterogeneous scaffolds in comparison with MSCs on homogeneous scaffolds at corresponding time points; Mean ± SD, *p* < 0.05

Next, we analyzed the metabolic state of the MSCs on the scaffolds at different stages after implantation. The FAD/NAD(P)H redox ratio in these MSCs in the case of the homogeneous scaffolds did not change significantly at any of the time points except for the 4th week, where we observed low values of this parameter (Fig. 8a, c).

For the heterogeneous scaffolds, it was shown that the FAD/NAD(P)H redox ratio in the MSCs did not change at any of the time points (Fig. 8b, c).

Using FLIM, we showed that the fluorescence lifetimes of the free and bound forms of NAD(P)H and FAD in MSCs for both types of scaffolds were higher than the standard values, which was probably due to the contribution from the background fluorescence of the scaffolds.

**Fig. 9:**
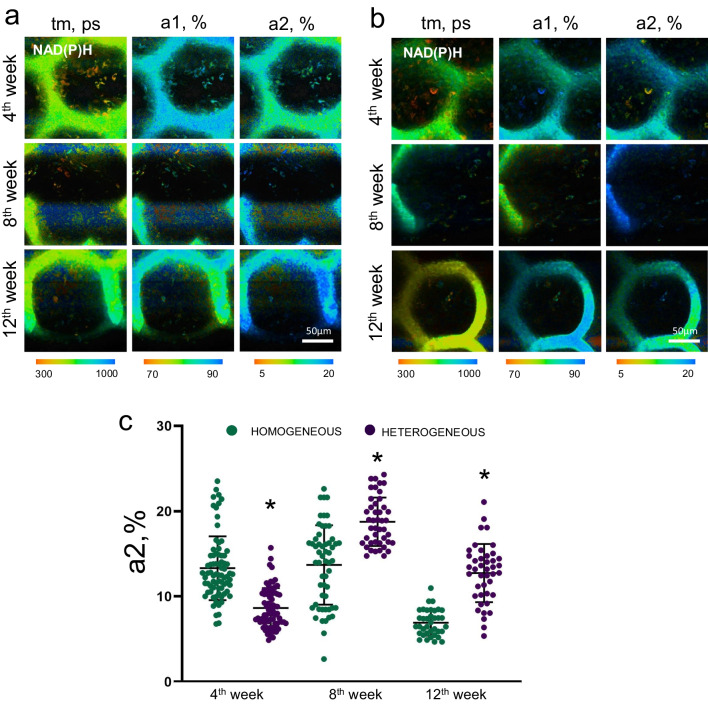
FLIM images of NAD(P)H in MSCs on **a** homogeneous and **b** heterogeneous scaffolds after implantation into the cranial bone; field of view 213 × 213 μm (512 × 512 pixels); × 400; **c** Fluorescence lifetime contribution of the bound form of NAD(P)H in MSCs on homogeneous and heterogeneous scaffolds after implantation; *statistically significant differences in MSCs on heterogeneous scaffolds in comparison with MSCs on homogeneous scaffolds at the corresponding time points; Mean ± SD, *p* < 0.05

By the 4th week after implantation the values of the fluorescence lifetime contributions of the bound form of NAD(P)H (a2) in the MSCs on the heterogeneous scaffolds were significantly lower than for the MSCs on homogeneous scaffolds. However, already on the 8th week, the a2 increased had significantly in the MSCs on heterogeneous scaffolds and had become higher than in the MSCs on homogeneous scaffolds. By the 12th week, the a2 in the MSCs on heterogeneous scaffolds decreased, however, the values were still significantly higher than in the MSCs on the homogeneous scaffolds at the same time point (Fig. 9a–c).

**Fig. 10:**
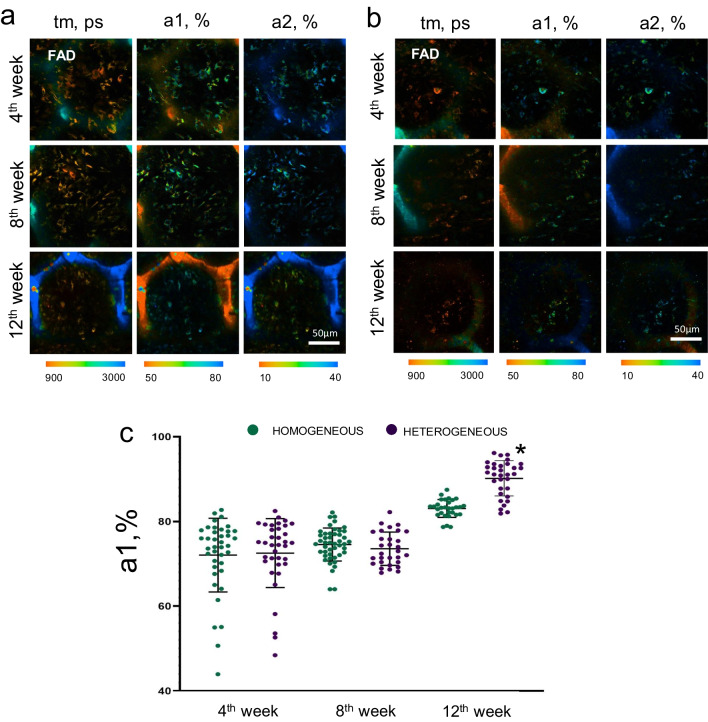
FLIM images of FAD in MSCs on **a** homogeneous and **b** heterogeneous scaffolds after implantation into the cranial bone; field of view 213 × 213 μm (512 × 512 pixels); × 400; **c** Fluorescence lifetime contribution of the bound form of FAD in MSCs on homogeneous and heterogeneous scaffolds after implantation; *statistically significant differences in MSCs on heterogeneous scaffolds in comparison with MSCs on homogeneous scaffolds at the corresponding time points; Mean ± SD, *p* < 0.05

The fluorescence contribution of the bound form of FAD (a1) in MSCs on both types of scaffold increased significantly by the 12th week after implantation, the increase was especially pronounced for the heterogeneous scaffolds (Fig. 10a–c).

A number of authors have shown that increases in the contributions of the bound form of NAD(P)H and the bound form of FAD are markers of stem cell differentiation in the osteogenic direction [32–34]. Therefore, the results obtained under in vivo conditions are consistent with our previous results, where we found that MSCs on heterogeneous scaffolds have a high rate of metabolic rearrangements associated with osteogenic differentiation.

#### Morphological analysis and Alizarin Red S staining

Morphological analysis made it possible to evaluate the integral complex structure including areas of undegraded scaffold and newly formed bone tissue.

**Fig. 11:**
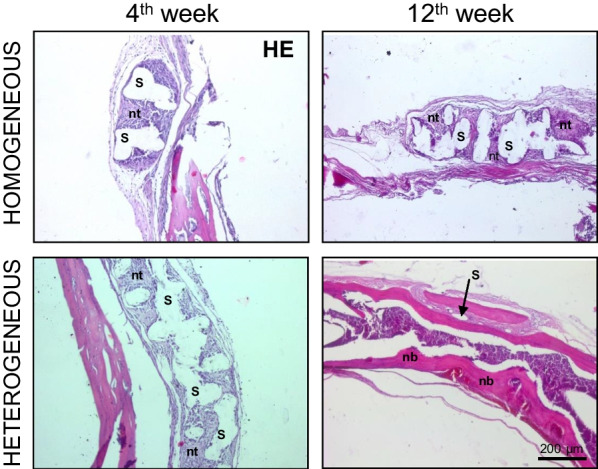
Histological analysis of bone tissue sections with implanted homogeneous and heterogeneous scaffolds; s, scaffold; nt, newly formed tissue; nb, newly formed bone; × 100

The morphological analysis of bone tissue samples with implanted scaffolds showed that by the 4th week in the case of homogeneous scaffolds, dense tissue had already been formed around the walls of the scaffold pores. However, even at the 12th week after implantation, the cells still exhibited similar morphological characteristics to undifferentiated MSCs (Fig. 11). At all time points, we can observe cavities in the developing tissue, which represent the walls of the scaffold pores that were destroyed at the stage of obtaining the histological sections.

By the 4th week after implantation, for the heterogeneous scaffolds, as in the case of the homogeneous scaffolds, we could observe areas of dense tissue formation that represented an accumulation of cells with the morphology of undifferentiated MSCs. However, at the 12th week after implantation, we revealed the formation of bone tissue with a spongy substance, having small areas of poorly differentiated cells (Fig. 11). At all time points, as in the case of the homogeneous scaffolds, we observed cavities that corresponded to inclusions of the scaffold polymer in the developing tissue. However, unlike the homogeneous scaffolds, the material of the heterogeneous scaffolds was almost completely replaced by bone tissue. Thus, for the heterogeneous scaffolds, a more efficient formation of bone tissue was demonstrated.

Thus, it can be concluded that heterogeneous scaffolds provide for the most effective MSC differentiation in the osteogenic direction and contribute to the most effective formation of bone tissue. This result is consistent with results obtained in vitro and once again confirms that scaffolds with a heterogeneous structure have high osteoinductive properties and provide for the effective formation of bone tissue.

## Discussion

The strategy of tissue engineering based on scaffolds with seeded MSCs is currently a promising approach for the treatment of extensive bone defects. At present, the influences of various characteristics of scaffolds on the behavior of cells and on the efficiency of the regenerative process have already been well described, such as the influence of the scaffold material itself, and its stiffness on cell infiltration, viability, migration and differentiation. One of the key issues is the determination of the optimal porosity of the scaffolds. It is known that a small pore size improves cell infiltration of the scaffolds and promotes better proliferation. However, small pore size does not facilitate cell migration into the scaffold and worsens cell aeration. Large pores, on the other hand, solve the problem of hindered cell migration, as well as improving the efficiency of differentiation, but the number of cells on scaffolds with a large pore size (more than 500 μm) is much lower, due to the loss of cells during cell seeding [35, 36]. This problem is partially solved by creating offset architecture (the optimal offset is 50/50) [37]. In this case, the cells have additional points for adhesion, thus improving cell density. However, homogeneous scaffolds with only one pore size are of limited use, as they do not completely mimic the native bone structure, since bone architecture is heterogeneous and has a pore size gradient going from dense to cancellous bone [38, 39]. Such a structure provides gradients in the tissues of oxygen, nutrients and active molecules, and therefore has a regulatory significance. Changes in gradient fluxes can affect the vital processes of cells, as well as their differentiation capacities. In this regard, the idea arose of creating scaffolds with a pore size gradient. Boccaccio et al. showed a more porous layer imitated light spongy cancellous bone, resulting in greater cell growth and transport of nutrients and waste in the highly porous regions [38].

However, until now there have been very few studies investigating the effect of scaffolds with a pore size gradient on the efficiency of MSC osteogenic differentiation. Moreover, most studies have largely been based on standard non-viable methods, such as the assessment of AP activity or staining with Alizarin Red S.

Abbasi et al. demonstrated the increased AP activity and superior matrix mineralization provided by heterogeneous scaffolds. However, after 30 days the AP activity decreased (compared to day 14), which does not fully correspond with our results. Also, the authors revealed a rise in the expression of elements of the signaling pathways that are involved in the process of osteogenic differentiation. In particular, the gradient scaffolds had a high level of *ocn*, *opn*, *bmp2* and *wnt5* gene expression [40]. Di Luca et al. showed that after 28 days in differentiation media AP activity was raised, moreover, the values reached a maximum in the zone of the large pores [41].

In our study, to evaluate the effect of scaffolds with a pore size gradient on the efficiency of MSC osteogenic differentiation we combined modern metabolic imaging and FLIM methods with standard methods.

We compared the efficiency of osteogenic differentiation of MSCs on scaffolds with a single pore size and on scaffolds with a heterogeneous pore size. As a result, it was revealed that heterogeneous scaffolds accelerate the process of osteogenic differentiation. Furthermore, the heterogeneous scaffolds have better osteoinductive properties and promote spontaneous osteogenic differentiation even in DMEM (control medium).

We based our findings on the fact that, during osteogenic differentiation, the metabolic state of the MSCs changes. Specifically, undifferentiated stem cells rely on glycolysis as an energy pathway, while, during differentiation, cells activate the OXPHOS system. This specific switch has been demonstrated for monolayer cultures by a number of authors [17, 33, 42].

The possibilities of the approach presented in this work for investigating stem cell metabolism based on the fluorescence features of NAD(P)H and FAD have already been demonstrated widely [43–47].

The optical redox ratio FAD/NAD(P)H is an informative metabolic profile indicator due to its strong correlation with the concentration ratio of NAD + /[NADH + NAD +] [48, 49]. If oxidative phosphorylation is enhanced in cells, the oxidation of NADH to NAD + and FADH2 to FAD + is prevalent over the glycolytic reduction of NAD + to NADH and so the redox ratio increases [20]. Specifically, decreases in the redox ratio have been reported for cells undergoing significant biosynthetic demands that require increased glucose catabolism, or in conditions like hypoxia in which oxidative phosphorylation is inhibited, but this is inconsistent with our data [49]. In our case, a significant decrease in the optical redox ratio was shown in the process of osteogenic differentiation of MSCs on both the homogeneous and heterogeneous scaffolds. A more rapid decline of this parameter was revealed particularly in MSCs on heterogeneous scaffolds. This result may be due to the fact that most of the studies based on analysis of the optical redox ratio have been performed on tumor cells, the energy metabolism of which is pretty primitive and based on glycolysis. In the case of stem cell differentiation, gradually activating the biosynthesis processes makes a great contribution to the NAD(P)H and FAD concentrations. For instance, Meleshina et al. showed that, despite a growth in the redox ratio in MSCs in the initial stage of osteogenic differentiation, there was a gradual decrease in this parameter at later stages [34]. Also, Quinn et al. found that the optical redox ratio initially decreased on week 1 in osteogenic cultures, following a slight increase in the redox ratio over time, but a still lower redox ratio was detected at week 4 relative to undifferentiated MSCs [50].

It is relevant to consider, that for the first time, we have performed the analysis of the metabolic imaging approach for MSCs on scaffolds (under 3D conditions), and the obtained results may therefore not fully correspond to the previous results for monolayer cultures. After all, various bone-graft substitutes influence cell metabolic activity in different manners [51].

It should be noted that methods based only on fluorescence characteristics do not provide useful information on the contributions of the various forms of NAD(P)H and FAD. Using the FLIM method, it is possible to separate the spectrally overlapping forms of these cofactors based on their fluorescence lifetimes. With NAD(P)H imaging, FLIM is typically used to determine the binding fraction of the fluorophores based on their unique lifetimes in their protein-bound and unbound states [30, 52]. FAD fluorescence lifetimes are sensitive to protein-binding and the presence of NAD + , however the biological interpretation of these measurements is not well understood [53]. The FLIM method allows us to assess the contribution of the metabolic pathways based on the fluorescence lifetime data, and the contributions of the various forms of NAD(P)H and FAD. The effectiveness of the FLIM method for assessing the metabolic state of MSCs (in monolayer culture) in the process of osteogenic differentiation has already been shown in a number of studies [34, 50, 54]. However, until now FLIM has not been used to assess the metabolic state of MSCs seeded on scaffolds. For both types of scaffold, during osteogenic differentiation, we revealed a rise in the fluorescence lifetime of the bound form of NAD(P)H (t2) probably associated with enhanced synthetic processes in the cells, in particular, the synthesis of extracellular matrix proteins. It was also shown that for both types of scaffold in DMEM and during osteogenic differentiation, there was an increase in the contributions of the bound form of NAD(P)H and the open form of FAD. This result indicates a growth in the contribution of oxidative phosphorylation in the process of osteogenic differentiation. It has been reported that OXPHOS becomes active only at the stage of osteogenic differentiation when proliferation is less pronounced, and this is in agreement with our data. Similar results were shown in the work of Meleshina et al. for monolayer MSC cultures, using optical redox ratio measurement and FLIM [34] where it was shown that there were increased values for the bound form of NAD(P)H and the open form of FAD, indicating a high intensity of OXPHOS as a characteristic feature in the process of the osteogenic differentiation of the MSCs. Chen et al. reported the activation of OXPHOS in MSCs during osteogenic differentiation, based on results from molecular analysis and alkaline phosphatase assay, as well as an evaluation of the lactate and ATP content and the level of oxygen consumption in the cells [32]. Guntur et al. performed bioenergetic profiling during osteogenic differentiation of calvarial osteoblasts and also observed the activation of OXPHOS [33]. In our work, particularly for heterogeneous scaffolds cultured in DMEM, there was a more pronounced increase in the contribution of the bound form of NAD(P)H by the 28th day. In the case of osteogenic differentiation, we also observed a more pronounced rise in the contribution of the bound form of NAD(P)H for the MSCs on heterogeneous scaffolds, than for those on homogeneous scaffolds. This confirms our conclusions that heterogeneous scaffolds promote more efficient osteogenic differentiation of MSCs.

Our results in vivo confirm that heterogeneous scaffolds provide for the formation of bone tissue, while in the case of homogeneous scaffolds, the mineralization, and formation of specific tissue morphology with spongy substance was not observed.

Based on the FAD/NAD(P)H optical redox ratio and FLIM analysis data, we observed a high contribution of the bound form of NADH (a2) at the later stages after implantation in MSCs on heterogeneous scaffolds, in comparison with MSCs on homogeneous scaffolds. As previously described in the case of the in vitro model, such high values of a2 indicate an increase in the intensity of OXPHOS as a marker of osteogenic differentiation [17, 55].

There are a number of works devoted to the study of the effectiveness of the restoration of bone defects using scaffolds made of poly(D,L-)lactide, however, the results are rather inconsistent. In particular, Li, et al. observed small amounts of bone tissue extending from the original bone to the PLGA homogeneous scaffolds, with large amounts of scaffold material remaining at 8 weeks post-implantation [56].

Barbieri et al. found that scaffolds based on poly(D,L-)lactide with the inclusion of nano-apatite showed the formation of immature bone tissue only at a high percentage of the nano-apatite component in the scaffold material, whereas, scaffolds with a low percentage of nano-apatite composites had a high rate of biodegradation, which prevented the formation of new bone tissue [57]. In contrast, Inui et al. showed that scaffolds based on a copolymer of poly(D,L-)lactide and Glycolide had dissolved and bone formation could be observed by the 8th week at the scaffold-bone interface [58]. Akagi et al. showed that that poly(D,L-)lactide scaffolds cause the formation of dense tissue at the implantation site, however, there is a significant increase in fibrous tissue, which replaces bone tissue [59]. In our work, we have shown that it is not only the material that affects the osteoinductive properties of the scaffolds, but the structure of the scaffolds, themselves, also plays a crucial role. And thus, it is possible to achieve a better efficiency of bone tissue formation only by creating a heterogeneous scaffold structure. Our conclusions are consistent with the data of other authors. Abbasi et al. showed significantly more newly-formed bone with the highest intensity of mineralization markers for a gradient porous architecture of polycaprolactone scaffolds, and confirmed significant enhancement of bone regeneration [60].

## Conclusion

Thus, in this work, using the modern label-free methods of multiphoton microscopy, in combination with FLIM, we performed a comprehensive analysis of the metabolic state of MSCs cultured on scaffolds with either a homogeneous or a heterogeneous structure in DMEM medium (control medium) and in a medium intended for the induction of osteogenic differentiation. As a result, heterogeneous scaffolds that mimic the native cancellous bone structure have been shown to provide improved osteoinductive properties and to accelerate the metabolic rearrangements associated with osteogenic differentiation. It is significant that the data obtained using these modern bioimaging methods are consistent with the results of standard methods for assessing the effectiveness of osteogenic differentiation of MSCs, such as alkaline phosphatase activity assay and Alizarin Red S staining. A comprehensive analysis of the efficiency of bone tissue formation under in vivo conditions showed that it is heterogeneous scaffolds that *best* provide for bone tissue recovery with specific tissue morphology, mineralization, and the formation of spongy substance.

## Supplementary Information

**Additional file 1**.** Table S1**. The primer sequences for RT-PCR.** Figure S1**. 3D model of the formed scaffold with a heterogeneous structure.** Table S2**. Evaluation of the viability of MSCs on scaffolds using calcein and propidium iodide staining.** Table S3**. Values of the NAD(P)H and FAD autofluorescence intensity, as well as the redox ratio FAD/NAD(P)H in MSCs on homogeneous and heterogeneous scaffolds during their cultivation in DMEM and in a medium for osteogenic differentiation.** Table S4**. FLIM data on NAD(P)H in MSCs on homogeneous and heterogeneous scaffolds during cultivation in DMEM and in a medium for the induction of osteogenic differentiation.** Table S5**. FLIM data of the FAD in MSCs on homogeneous and heterogeneous scaffolds during cultivation in DMEM and in a medium for the induction of osteogenic differentiation.** Figure S2**. An example of the force versus indentation curve together with the Hertz’s model fit (fit of the approach part of the curve).** Figure S3**. (a) Autofluorescence and optical redox ratio images of NAD(P)H and FAD in MSCs on homogeneous and heterogeneous scaffolds before implantation. (b) analysis of the viability of MSCs on scaffolds, stained with calcein and propidium iodide, cell nuclei stained with Hoekst; (c) fluorescence lifetime contribution of the bound form of NAD(P)H in MSCs on homogeneous and heterogeneous scaffolds before implantation.** Figure S4**. FLIM images of (a) NAD(P)H and (c) FAD in MSCs on homogeneous and heterogeneous scaffolds before implantation;(с) Fluorescence lifetime contributions of the bound form of (b) NAD(P)H and (d) FAD in MSCs on homogeneous and heterogeneous scaffolds before implantation.** Figure S5**. Evaluation of multiple changes in the expression of gene-markers of osteogenesis relative to the first day of cultivation of MSCs on homogeneous scaffolds; cultivation in (a) control medium and (b) osteogenic medium.

## Data Availability

All data generated or analyzed during this study are included in this published article and supplementary materials.

## Abbreviations

AP: Alkaline phosphatase
ATP: Adenosine triphosphate
FAD: Flavin adenine dinucleotide
FLIM: Fluorescence lifetime imaging microscopy
MSCs: Mesenchymal stem cells
NAD(P)H: Nicotinamide adenine dinucleotide
OXPHOS: Oxidative phosphorylation
