# Supplementary material for: FLIM imaging revealed spontaneous osteogenic differentiation of stem cells on gradient pore size tissue-engineered constructs
Source: Stem Cell Res Ther. 2023 Apr 12;14:81. doi: 10.1186/s13287-023-03307-6 (PMC10091689; doi:10.1186/s13287-023-03307-6)
Supplement: Supplementary file 1 — Additional file 1. Table S1. The primer sequences for RT-PCR. Figure S1. 3D model of the formed scaffold with a heterogeneous structure. Table S2. Evaluation of the viability of MSCs on scaffolds using calcein and propidium iodide staining. Table S3. Values of the NAD(P)H and FAD autofluorescence intensity, as well as the redox ratio FAD/NAD(P)H in MSCs on homogeneous and heterogeneous scaffolds during their cultivation in DMEM and in a medium for osteogenic differentiation. Table S4. FLIM data on NAD(P)H in MSCs on homogeneous and heterogeneous scaffolds during cultivation in DMEM and in a medium for the induction of osteogenic differentiation. Table S5. FLIM data of the FAD in MSCs on homogeneous and heterogeneous scaffolds during cultivation in DMEM and in a medium for the induction of osteogenic differentiation. Figure S2. An example of the force versus indentation curve together with the Hertz’s model fit (fit of the approach part of the curve). Figure S3. (a) Autofluorescence and optical redox ratio images of NAD(P)H and FAD in MSCs on homogeneous and heterogeneous scaffolds before implantation. (b) analysis of the viability of MSCs on scaffolds, stained with calcein and propidium iodide, cell nuclei stained with Hoekst; (c) fluorescence lifetime contribution of the bound form of NAD(P)H in MSCs on homogeneous and heterogeneous scaffolds before implantation. Figure S4. FLIM images of (a) NAD(P)H and (c) FAD in MSCs on homogeneous and heterogeneous scaffolds before implantation;(с) Fluorescence lifetime contributions of the bound form of (b) NAD(P)H and (d) FAD in MSCs on homogeneous and heterogeneous scaffolds before implantation. Figure S5. Evaluation of multiple changes in the expression of gene-markers of osteogenesis relative to the first day of cultivation of MSCs on homogeneous scaffolds; cultivation in (a) control medium and (b) osteogenic medium. [file 13287_2023_3307_MOESM1_ESM.docx]

**Supplementary materials**

**FLIM imaging revealed spontaneous osteogenic differentiation of stem cells on gradient pore size tissue engineered constructs**

*Svetlana Rodimova^1,2^, Artem Mozherov^2^, Vadim Elagin^2^, Maria Karabut^2^, Ilya Shchechkin^1,2^, Dmitry Kozlov^1,2^, Dmitry Krylov^1,2^, Alena Gavrina^1,2^, Vladislav Kaplin^3^, Evgenii Epifanov^4^, Nikita Minaev^5^, Ksenia Bardakova^4,5^, Anna Solovieva^3^, Peter Timashev^5,6^, Elena Zagaynova^1,2^, Daria Kuznetsova^1,2^.*

1. N.I. Lobachevsky Nizhny Novgorod National Research State University, Nizhny Novgorod, 23 Gagarina ave., 603022, Russia
2. Institute of Experimental Oncology and Biomedical Technologies, Privolzhsky Research Medical University, 10/1 Minin and Pozharsky sq., Nizhny Novgorod, 603000, Russia
3. Semenov Federal Research Center of Chemical Physics, Russian Academy of Sciences, 4 Kosygina St, Moscow, 119991, Russia
4. Institute of Photonic Technologies, Research Center “Crystallography and Photonics”, Russian Academy of Sciences, 2 Pionerskaya St, Troitsk, Moscow, 108840, Russia
5. Institute for Regenerative Medicine, Sechenov University, 8-2 Trubetskaya str, Moscow, 119991, Russia
6. World-Class Research Center "Digital biodesign and personalized healthcare", Sechenov University, 8-2 Trubetskaya str, Moscow, 119991, Russia

E-mail: [srodimova123@gmail.com](mailto:srodimova123@gmail.com)

**Table S1.** The primer sequences for RT-PCR.

| **Primer Target** | **Primer Sequence (5′→3′)** |
| --- | --- |
| OPG | F:GGTCTCCTGCTAACTCAGAAAGG |
|  | R:CAGCAAACCTGAAGAATGCCTCC |
| SDF1 | F:CTCAACACTCCAAACTGTGCCC |
|  | R:CTCCAGGTACTCCTGAATCCAC |
| COL1A1 | F:CCTGGCTTTCTCGTCACTCTCA |
|  | R:AGCCTCTCCATCTTTGCCAGCA |
| RUNX2 | F: CCCAGTATGAGAGTAGGTGTCC |
|  | R: GGGTAAGACTGGTCATAGGACC |
| SPARC | F:TGCCTGATGAGACAGAGGTGGT |
|  | R:CTTCGGTTTCCTCTGCACCATC |
| HPRT1 | F:TCAAGGGCATATCCTACAACAA |
|  | R:AGACTTTGCTTTCCTTGGTCAG |


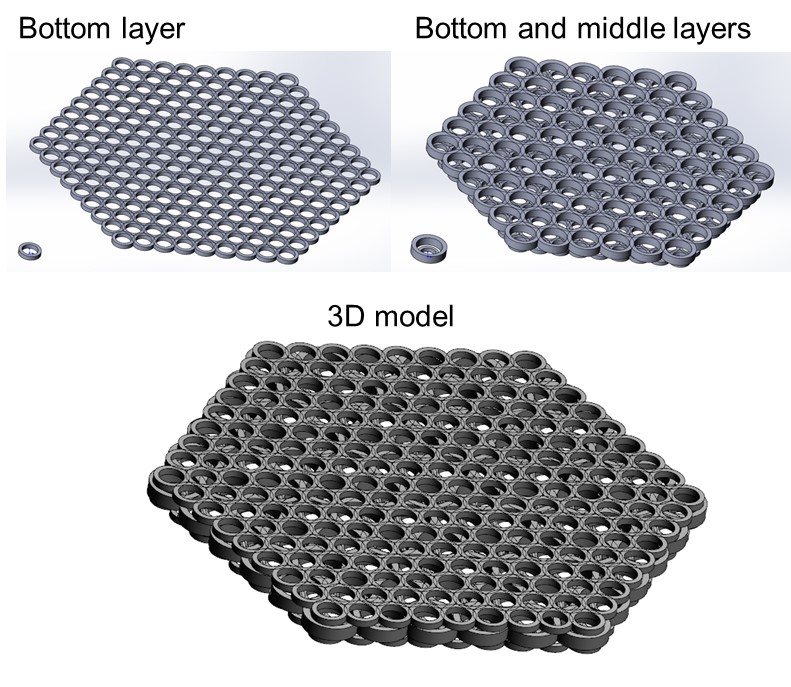


**Figure S1.** 3D model of the formed scaffold with a heterogeneous structure.

**Table S2.** Evaluation of the viability of MSCs on scaffolds using calcein and propidium iodide staining


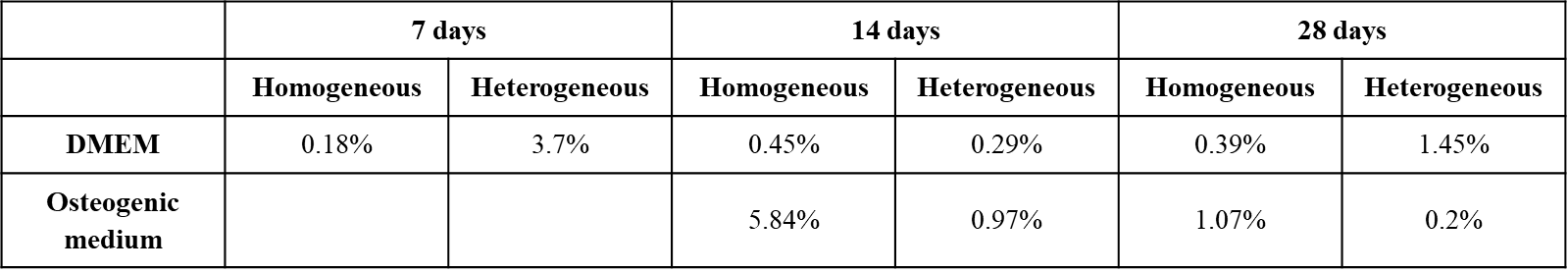


Values are presented as the percentage of dead cells relative to the total number of cells on the scaffold in a x40 field of view.

**Table S3.** Values of the NAD(P)H and FAD autofluorescence intensity, as well as the redox ratio FAD/NAD(P)H in MSCs on homogeneous and heterogeneous scaffolds during their cultivation in DMEM and in a medium for osteogenic differentiation.


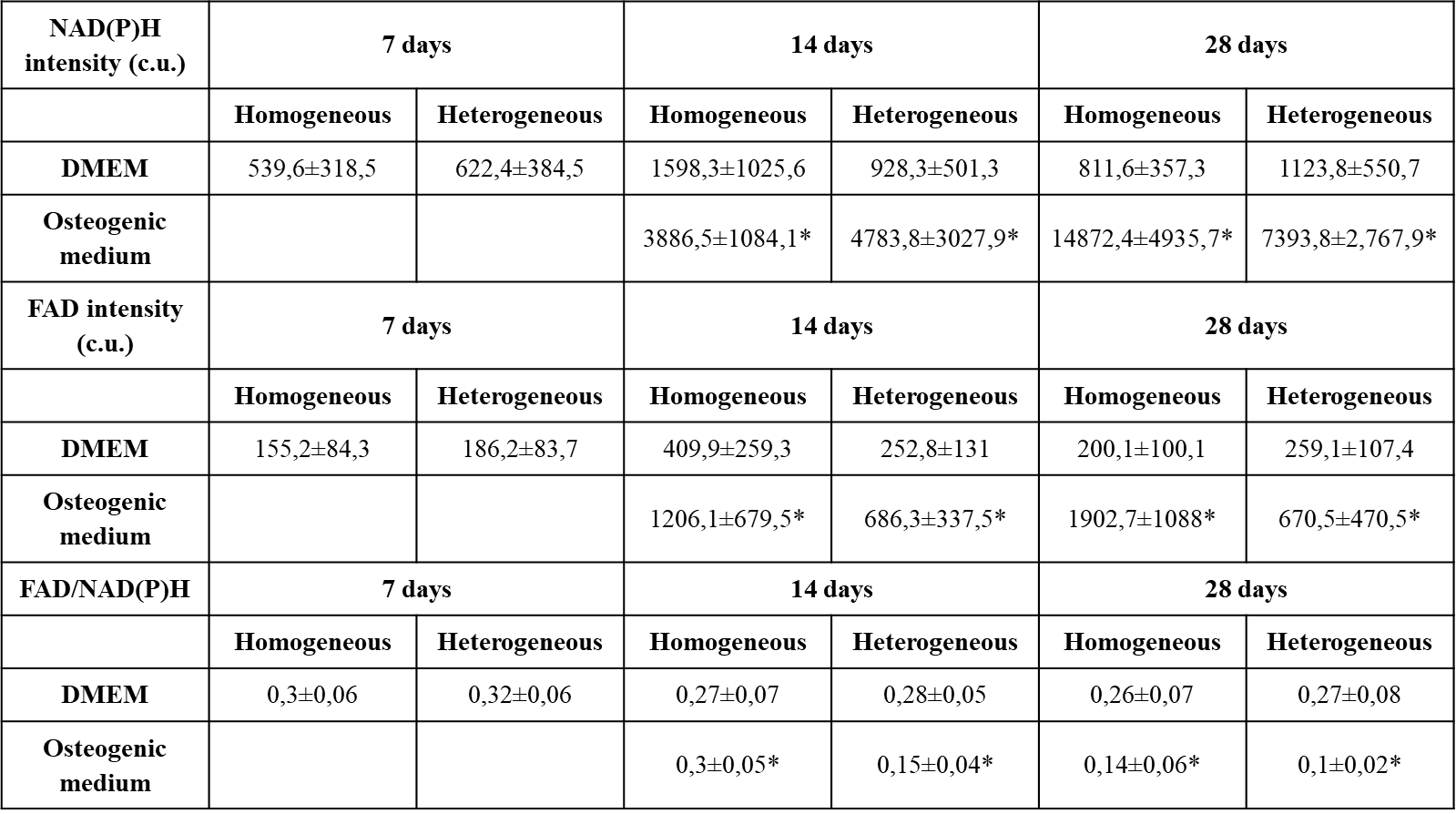


* — statistically significant differences in comparison with MSCs on the 7th day of cultivation in DMEM; Mean ± SD, p <0.05.

**Table S4.** FLIM data on NAD(P)H in MSCs on homogeneous and heterogeneous scaffolds during cultivation in DMEM and in a medium for the induction of osteogenic differentiation.

**
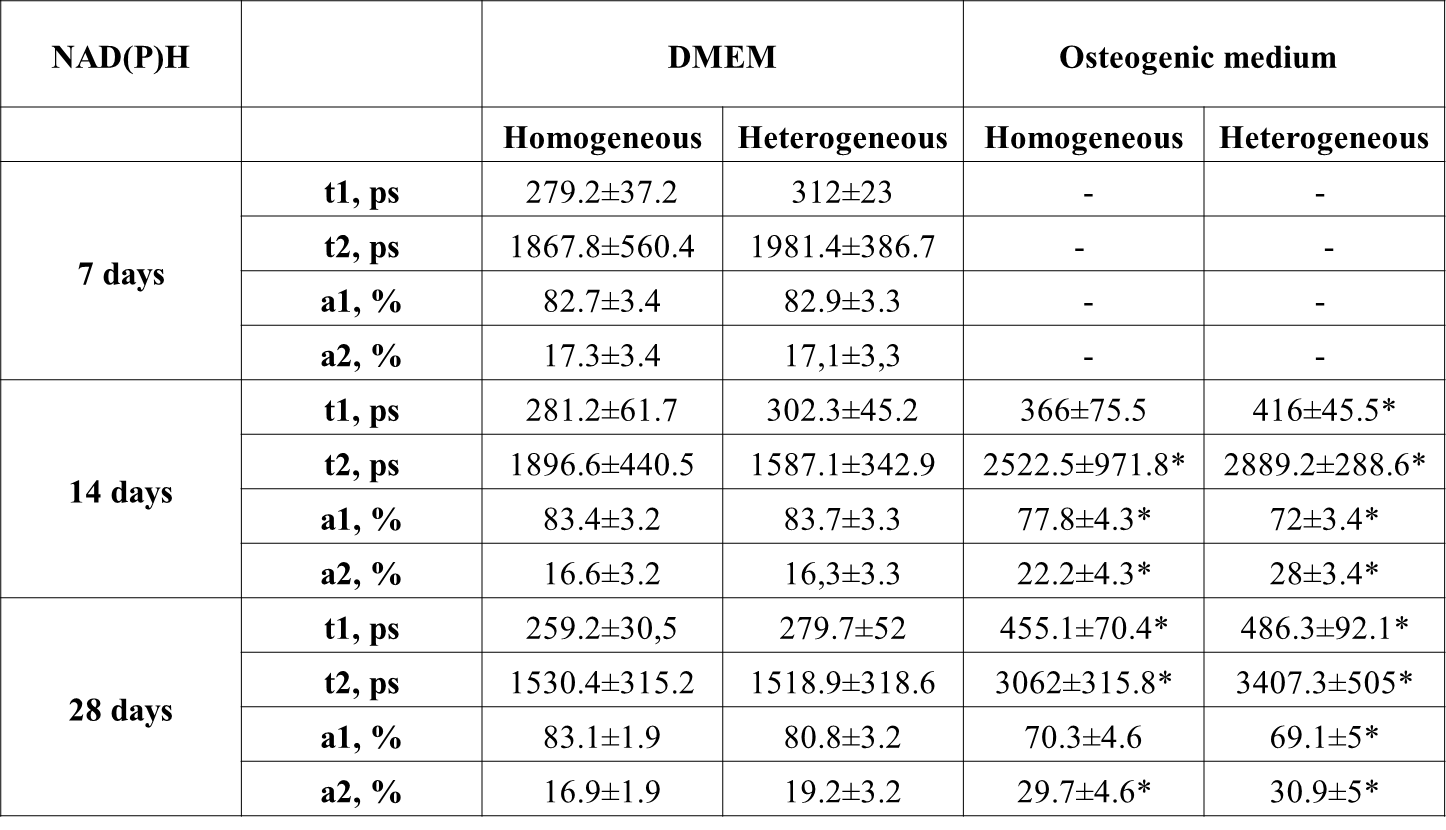
**

**Table S5.** FLIM data of the FAD in MSCs on homogeneous and heterogeneous scaffolds during cultivation in DMEM and in a medium for the induction of osteogenic differentiation.


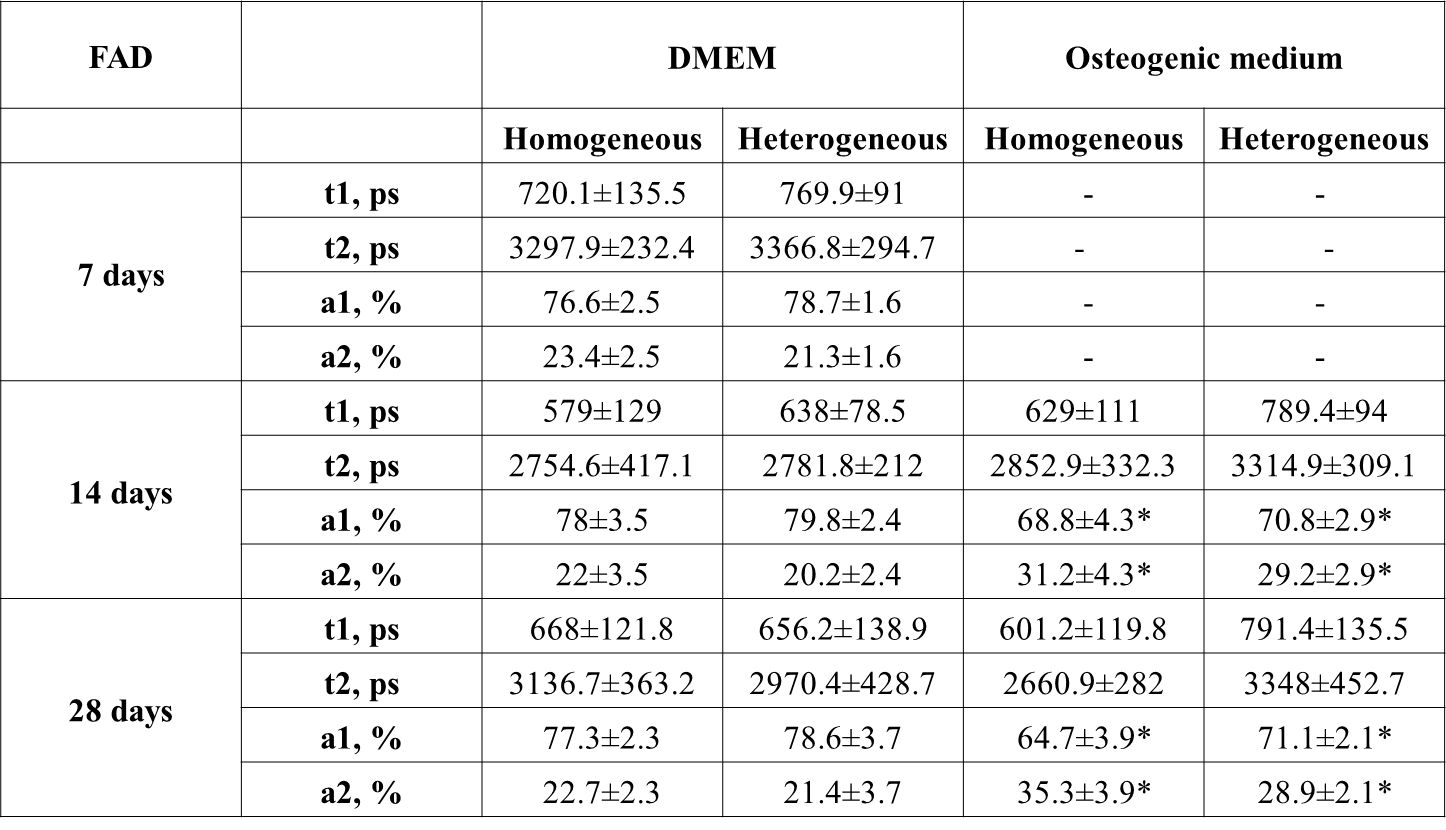


**
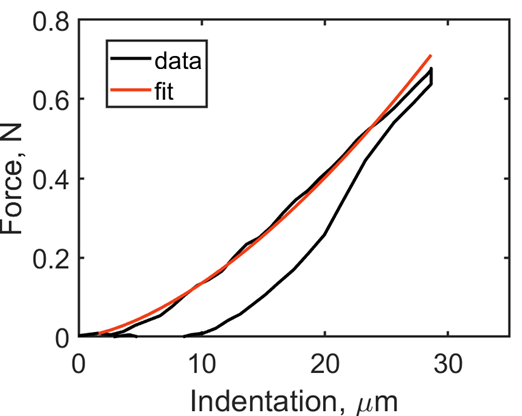
**

**Figure S2.** An example of the force versus indentation curve together with the Hertz’s model fit (fit of the approach part of the curve).


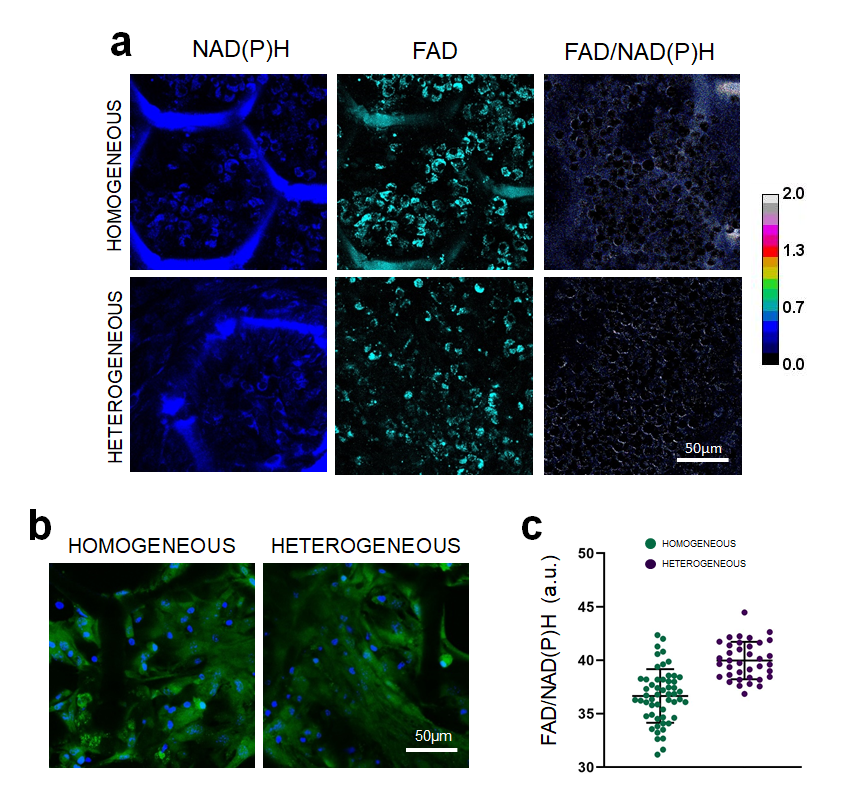


**Figure S3. (a)** Autofluorescence and optical redox ratio images of NAD(P)H and FAD in MSCs on homogeneous and heterogeneous scaffolds before implantation. Field of view 213 × 213 µm (1024 × 1024 pixels); x400; **(b)** analysis of the viability of MSCs on scaffolds, stained with calcein and propidium iodide, cell nuclei stained with Hoekst; x400;**( c)** fluorescence lifetime contribution of the bound form of NAD(P)H in MSCs on homogeneous and heterogeneous scaffolds before implantation; Mean±SD.


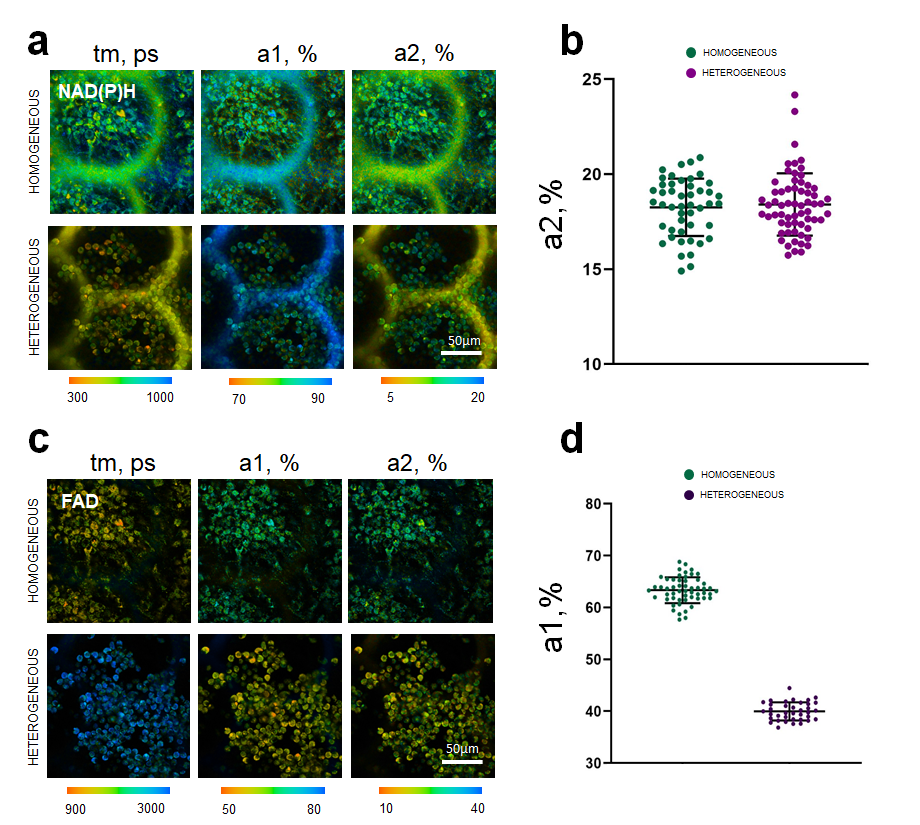


**Figure S4.** FLIM images of **(a)** NAD(P)H and **(c)** FAD in MSCs on homogeneous and heterogeneous scaffolds before implantation; field of view 213 × 213 μm (512 × 512 pixels); x400; **(с)** Fluorescence lifetime contributions of the bound form of **(b)** NAD(P)H and **(d)** FAD in MSCs on homogeneous and heterogeneous scaffolds  before implantation; Mean±SD.

*Real-time PCR*

To evaluate the expression profiles of MSCs cultivated on scaffolds, we obtained data using Real-time RT-PCR. Runx2 is an important transcription factor for osteoblast differentiation, matrix formation, and mineralization during bone formation because Runx2 regulates downstream genes that determine the osteoblast phenotype and function in the early stages of osteogenesis [1]. On the 28th day of cultivation in control medium, we observed a sharp increase in the level of RUNX2 in MSCs on the heterogeneous scaffolds (by 6.1 times (p-value ≤ 0.05) compared to those on homogeneous scaffolds). Such an increase in expression may indicate spontaneous induction of osteogenic differentiation due to the osteogenic properties of the scaffold (Figure S5a). In MSCs on both types of scaffolds cultured in osteogenic medium, we did not observe any increase in the RUNX2 level although that lack of apparent change during the experimental sampling period could have been due to an early activation of expression of this gene, that we were unable to capture. (Figure S5b).


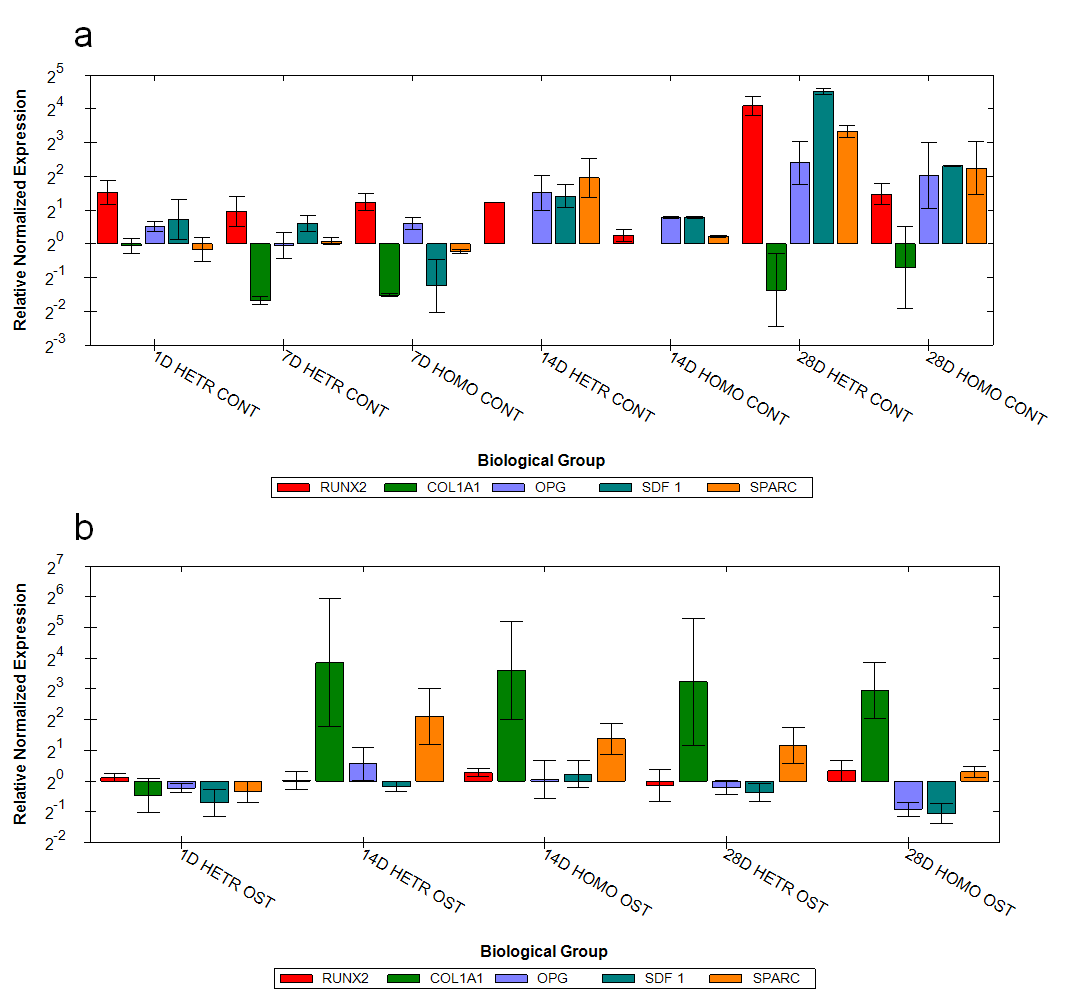


**Figure S5.** Evaluation of multiple changes in the expression of gene-markers of osteogenesis relative to the first day of cultivation of MSCs on homogeneous scaffolds; cultivation in (a) control medium and (b) osteogenic medium.

Collagen is the main component of the extracellular matrix, with type I collagen making up 90% of the organic matter of the bone matrix, it is also one of the key components of the MSC cell niche [2]. During osteogenic differentiation, we could already observe a sharp up-regulation of COL1A1 by the 14th day of MSC cultivation on both types of scaffold, this trend persisting up to the 28th day (Figure S5b), which is consistent with the literature data [3,4].

Osteoprotegerin (OPG) is a soluble decoy receptor for RANKL and prevents RANKL from binding to RANK, thereby inhibiting osteoclast differentiation. Osteoblasts regulate osteoclast differentiation by secreting and expressing OPG and RANKL [5]. It was shown that in MSCs on scaffolds cultivated in the control medium, a stable, increasing expression level of OPG occurred for both the homogeneous and heterogeneous scaffolds during cultivation (Figure S4a). During osteogenic differentiation, we also observed up-regulated OPG up to the 28th day, it being most pronounced for the heterogeneous scaffolds (1.7 times compared to the homogeneous scaffolds on the 28th day of cultivation, p-value ≤ 0.05) (Figure S5b). This is a favorable factor for increasing bone mass and bone regeneration [6].

In MSCs on scaffolds cultivated in the control medium, a gradual increase in the level of SDF-1 expression was shown, it being more actively expressed by undifferentiated MSCs [7] and is associated with the formation of intercellular contacts, providing for their homing and proliferation [8] (Figure S5a). At the same time, for MSCs on both types of scaffold cultivated in osteogenic medium, we revealed a sharp decrease in the level of SDF expression by the 28th day (1.25 and 2.5-fold (p-value ≤ 0.05) for the homogeneous and heterogeneous scaffolds, respectively. compared to the 14th day of cultivation) (Figure S5b).

Osteonectin (SPARC) is a protein that regulates the activity of matrix metalloproteinases, collagen laying, and its early-stage mineralization [9,10]. It is also possibly involved in regulation of the VEGF, TGF-β1 and FGF signaling pathways [11,12]. Heterogeneous scaffolds were characterized by an earlier increase in the expression SPARC (on the 14th day of cultivation in the control medium, its expression being 3.3 times (p-value ≤ 0.05) higher than for the MSCs on homogeneous scaffolds). We also showed a sharp increase in the expression of SPARC by the 28th day of MSC cultivation in the control medium for both types of scaffold (4 times and 2.3 times (p-value ≤ 0.05) increase for homogeneous and heterogeneous scaffolds respectively, compared to the 14th day of cultivation) (Figure S5a). In the process of osteogenic differentiation, we revealed a gradual decrease in the expression of SPARC by the 28th day of cultivation on both types of scaffold (2 times and 1.9 times (p-value ≤ 0.05) for the homogeneous and heterogeneous scaffolds, respectively, compared with the 14th day of cultivation) (Figure S5b), which is consistent with the literature data [13]. During the late stages of cultivation, SPARC expression was slightly higher for the heterogeneous scaffolds (by 1.7 and 1.8 times on 14th and 28th days of cultivation relative to the MSCs on homogeneous scaffolds) (Figure S5b).

**References**

[1] Li J, Xu Q, Teng B, Yu C, Song L, Lai YX, et al. Investigation of angiogenesis in bioactive 3-dimensional poly (d, l-lactide-co-glycolide)/nano-hydroxyapatite scaffolds by in vivo multiphoton microscopy in murine calvarial critical bone defect. Acta Biomater. 2016; 4: 389-399.

[1] Komori T. Regulation of bone development and extracellular matrix protein genes by RUNX2. Cell Tissue Res. 2010;. 339: 189-195.

[2] Delmas VCSGP. PD The role of collagen in bone strength. Osteoporos Int, 2006; 17: 319-36.

[3] Twine NA, Chen L, Pang CN, Wilkins MR, Kassem M. Identification of differentiation-stage specific markers that define the ex vivo osteoblastic phenotype. Bone. 2014; 67: 23-32.

[4] Kulterer B, Friedl G, Jandrositz A, Sanchez-Cabo F, Prokesch A, Paar C. et al. Gene expression profiling of human mesenchymal stem cells derived from bone marrow during expansion and osteoblast differentiation. BMC genomics. 2007; 8: 1-15.

[5] Han X, Gong S, Li N, Wang X, Liu P, Xu Y. et al. A novel small molecule which increases osteoprotegerin expression and protects against ovariectomy-related bone loss in rats. Front Pharmacol. 2019; 10: 103.

[6] Cawley KM, Bustamante-Gomez NC, Guha AG, MacLeod RS, Xiong J, Gubrij I. 2020. Local production of osteoprotegerin by osteoblasts suppresses bone resorption. Cell Reports. 2020; 32: 108052.

[7] Ding L, Morrison SJ. Haematopoietic stem cells and early lymphoid progenitors occupy distinct bone marrow niches. Nature. 2013; 495: 231-235.

[8] Schajnovitz A, Itkin T, D'uva G, Kalinkovich A, Golan K, Ludin A. et al. CXCL12 secretion by bone marrow stromal cells is dependent on cell contact and mediated by connexin-43 and connexin-45 gap junctions. Nat Immunol. 2011; 12: 391-398.

[9] Twine NA, Chen L, Pang CN, Wilkins MR, Kassem M. Identification of differentiation-stage specific markers that define the ex vivo osteoblastic phenotype. Bone. 2014; 67: 23-32.

[10] Bradshaw AD. Diverse biological functions of the SPARC family of proteins. The international journal of biochemistry & cell biology. 2012; 44: 480-488.

[11] Wang Q, Yang Q, Zhang A, Kang Z, Wang Y, et al. Silencing of SPARC represses heterotopic ossification via inhibition of the MAPK signaling pathway. Bioscience reports. 2019; 39.

[12] Rivera LB, Bradshaw AD, Brekken RA. The regulatory function of SPARC in vascular biology. Cellular and Molecular Life Sciences. 2011; 68: 3165-3173.

[13] Twine NA, Chen L, Pang CN, Wilkins MR, Kassem, M. Identification of differentiation-stage specific markers that define the ex vivo osteoblastic phenotype. Bone. 2014; 67: 23-32.
